# Radioresistance Mechanisms in Prostate Cancer Cell Lines Surviving Ultra-Hypo-Fractionated EBRT: Implications and Possible Clinical Applications

**DOI:** 10.3390/cancers14225504

**Published:** 2022-11-09

**Authors:** Silvia Sideri, Francesco Petragnano, Roberto Maggio, Simonetta Petrungaro, Angela Catizone, Luisa Gesualdi, Viviana De Martino, Giulia Battafarano, Andrea Del Fattore, Domenico Liguoro, Paola De Cesaris, Antonio Filippini, Francesco Marampon, Anna Riccioli

**Affiliations:** 1 Department of Anatomy, Histology, Forensic Medicine and Orthopaedics, Section of Histology and Medical Embryology, “Sapienza” University, 00161 Rome, Italy; 2 Department of Biotechnological and Applied Clinical Sciences, University of L’Aquila, 67100 L’Aquila, Italy; 3 Bone Physiopathology Research Unit, Genetics and Rare Diseases Research Division, Bambino Gesù Children’s Hospital, IRCCS, 00146 Rome, Italy; 4 Department of Clinical and Molecular Medicine, Sant’Andrea Hospital, “Sapienza” University, 00185 Rome, Italy; 5 Department of Life, Health and Environmental Sciences, University of L’Aquila, 67100 L’Aquila, Italy; 6 Department of Radiotherapy, “Sapienza” University of Rome, 00161 Rome, Italy

**Keywords:** prostate cancer, radiotherapy, bone metastasis, brain metastasis, EMT, docetaxel

## Abstract

**Simple Summary:**

Radiotherapy is an option for curing localized and locally advanced prostate cancer. However, radioresistance can occur, determining treatment failure and poor prognosis. Herein, we developed a model of radio-resistant prostate cancer cells by irradiating the bone metastasis-derived PC3 highly metastatic prostate cancer cell line and the brain-derived moderately metastatic DU-145 prostate cancer cell line, both castration-resistant. Ultra-hypo-fractionated radiotherapy was used, with doses and intervals similar to the ones used in clinical practice. These in vitro models were tested to gain information on the molecular mechanisms used by prostate cancer cells to survive radiation-induced death. Results from bioassays and molecular assays show that in the highly metastatic cells (PC3), the acquired radioresistance—though enhancing clonogenic efficiency, enrichment of cancer stem cells, proliferation rate and migration ability—interestingly results in significantly higher sensitivity to Docetaxel. This behaviour was not observed using the moderately metastatic DU-145 prostate cancer cells. It can be hypothesised that subgroups of patients with highly metastatic prostate cancer could benefit from chemotherapy immediately after the failure of radiotherapy, before a re-challenge with hormonal treatment or other strategies.

**Abstract:**

The use of a higher dose per fraction to overcome the high radioresistance of prostate cancer cells has been unsuccessfully proposed. Herein, we present PC3 and DU-145, castration-resistant prostate cancer cell lines that survived a clinically used ultra-higher dose per fraction, namely, radioresistant PC3 and DU-145 cells (PC3RR and DU-145RR). Compared to PC3, PC3RR showed a higher level of aggressive behaviour, with enhanced clonogenic potential, DNA damage repair, migration ability and cancer stem cell features. Furthermore, compared to PC3, PC3RR more efficiently survived further radiation by increasing proliferation and down-regulating pro-apoptotic proteins. No significant changes of the above parameters were described in DU-145RR, suggesting that different prostate cancer cell lines that survive ultra-higher dose per fraction do not display the same grade of aggressive phenotype. Furthermore, both PC3RR and DU-145RR increased antioxidant enzymes and mesenchymal markers. Our data suggest that different molecular mechanisms could be potential targets for future treatments plans based on sequential strategies and synergistic effects of different modalities, possibly in a patient-tailored fashion. Moreover, PC3RR cells displayed an increase in specific markers involved in bone remodeling, indicating that radiotherapy selects a PC3 population capable of migrating to secondary metastatic sites. Finally, PC3RR cells showed a better sensitivity to Docetaxel as compared to native PC3 cells. This suggests that a subset of patients with castration-resistant metastatic disease could benefit from upfront Docetaxel treatment after the failure of radiotherapy.

## 1. Introduction

Prostate cancer (PCa) is the second most frequent malignancy in men worldwide, counting more than one million new cases and causing three hundred thousand deaths, thus representing 6.8% of all deaths caused by cancer in men in 2020 [1]. Current treatment for PCa—depending on several factors, such as the patient’s risk class, age and performance status—includes surgery and/or radiotherapy (RT) [2], with surgery being the most chosen treatment option [3]. Conventional RT treatment is based on the use of daily fractions of 2 Gy, up to 70–80 Gy in total dose, in combination with androgen deprivation therapy or not (ADT) [2]. Due to technological advances enhancing the ability of RT to deliver a higher dose of ionizing radiation (IR) with greater precision [4,5], as well as the progressive reduction of the costs, an increased use of RT for treating PCa is expected [6]. Moreover, in a multimodal approach to reduce the risk of local relapse or systemic spread of the disease, post-operative RT is an option in selected cases, although the optimal timing of RT after radical prostatectomy remains debated [7]. Approximately 30% of irradiated PCa patients experience a biochemical recurrence [8], often associated with the presence of distant metastases [9]. In patients with PCa, bone represents 90% of the metastatic sites, within which the spine is the most frequent. Metastatic spread and biological characteristics of metastasis in PCa patients are of clinical relevance to RT response [10]. Indeed, to overcome the potential relapse of cancer cells, the use of a higher dose per fraction has been proposed, namely hypo-fractionated RT (HFRT) [11] that could favour cancer cell death [12]. However, clinical experiences have not reported any significant difference between HFRT, ultra-HFRT and conventionally-fractionated RT [13,14], indicating that increasing the dose could be insufficient to overcome PCa radioresistance. Thus, to improve the therapeutic efficacy of HFRT, it is crucial to elucidate the molecular mechanisms sustaining intrinsic and acquired cellular radioresistance, in order to identify new radiosensitizing strategies [12,15].

RT induces cancer cell death by directly or indirectly promoting DNA damage, such as double-strand breaks (DSBs), through the generation of reactive oxygen species (ROS) [16]. Cancer cells, including PCa, can escape from RT-induced cell death by activating different molecular mechanisms, such as deregulated DNA repair, ROS detoxification, activation of cell receptors and related downstream signal transduction pathways [17,18,19]. Moreover, it has been reported that RT can reprogram radiosensitive cancer cells into radioresistant cancer cells through a process named cellular plasticity [20]. In this regard, it has been shown that conventional RT could determine the selection and enrichment of PCa cancer-stem-like cells (CSCs), the major contributors to radioresistance [21,22] and metastasis onset [23], through the activation of epithelial–mesenchymal transition (EMT) [24,25,26].

Notably, most of the studies investigating cancer cells radioresistance mechanisms used cancer cells with a different grade of intrinsic radioresistance and genetic background. These features do not allow us to obtain biologically solid results [27,28,29]. Recently, several isogenic PCa models of radioresistance were selected after repeated exposure to conventional fractionation of IR [30,31], but no studies have been published so far on PCa resistance to HFRT.

In this study, we present two new models of isogenic radioresistant PCa cell lines, selected by using an ultra-HFRT schedule. To this purpose, castration-resistant PC3 and DU-145, respectively, highly and moderately aggressive PCa cell lines [32], were irradiated with six fractions of 6 Gy each, resembling the protocol used with PCa patients in current clinical practice [33]. The surviving radioresistant cells, PC3RR, DU-145RR and their respective isogenic parental cell lines, PC3 and DU-145, were used for several bio- and molecular assays. Our data seem to suggest that resistance to high doses of radiation is associated with cellular changes such as aggressive basal behaviour, in an interestingly cell-type-dependent manner. This information might be translated into meaningful clinical applications in order to improve the therapeutic efficiency of RT.

## 2. Materials and Methods

### 2.1. Cell Lines and Reagents

PC3 cells (Lot: 61777391) and DU-145 cells (Lot: 59722255) were obtained from the American Type Culture Collection (ATCC, Manassas, VA, USA), cultured in RPMI-1640 and Eagle Minimum Essential Medium(EMEM) (Sigma, Saint Louis, MO, USA), respectively, supplemented with 2 mM of L-glutamine (Sigma), 200 U/mL of penicillin–streptomycin (Sigma), 1 mM of sodium pyruvate (Sigma), 10 mM of Hepes (Sigma) and 10% fetal bovine serum (FBS) (Life Technologies-Gibco, Eugene, OR, USA). Cells were maintained at 37 °C in a humidified 5% CO_2_ incubator and routinely checked for mycoplasma. For anchorage-independence experiments, cells were cultured in ultralow attachment flasks (Corning Kennebunk, ME, USA) with complete culture medium.

### 2.2. Irradiation of PC3 and DU-145 Cells and Clonogenic Assay

Radiation was delivered at room temperature using an x-6 MV photon linear accelerator. The total single dose was delivered with a dose rate of 2 Gy/min using a source-to-surface distance (SSD) of 100 cm. The doses were of 200 kV X-rays (Yxlon Y.TU 320; Yxlon, Copenhagen, Denmark) filtered with 0.5 mm Cu. The absorbed dose was measured using a Duplex dosimeter (PTW, Freiburg, Germany). To select clinically relevant radioresistant (RR) cell lines, 24 h after irradiation, 30% of irradiated cells were re-seeded and the next irradiation was repeated when a confluence of 80% was reached again. This was repeated for 6 times following the hypofractionated schedule used for treating PCa patients (α/β ratio for PCa = 1.5, Biologically Effective Dose-BED = 180 Gy) [34].

For the subsequent treatments of isogenic PCa cell lines, PC3RR cells and DU-145RR, radiation was delivered at room temperature using a 6 MV X-ray linear accelerator. Every single dose of X-ray radiation was delivered at a distance of 100 cm from the surface of the source. Control cells were managed identically to the irradiated cells, with the exception of radiation exposure. The clonogenic assay was performed on both cell lines and treated with increasing doses of radiation (2 to 8 Gy). After 3 h from radiation, 400–800 cells per well were plated, while the control untreated cells were plated with 100–200 in each well. After 11 days, cells were fixed with 4% paraformaldehyde for 15 min at room temperature (RT) and stained with Crystal Violet. The average colony count for the treated and untreated cells was performed using the ImageJ program, and the number of colonies counted was used to calculate plating efficiency for the control cells and the surviving fraction for the irradiated cells, as described [35].

### 2.3. Western Blot Analysis

Total cell protein extraction was performed by homogenizing cells in lysis buffer containing protease and phosphatase inhibitor cocktail (Cell Signaling, Danvers, MA, USA). After sonication, the homogenates were centrifuged at 400× *g* rpm for 15 min at 4 °C. Protein concentration was determined with a Bicinchoninic acid assay (BCA) (Pierce, Rockford, IL, USA). Protein lysates obtained from the cells were separated on SDS-PAGE gel cells and transferred onto nitrocellulose membranes (Amersham Bioscience, Little Chalfont, UK). The filters were saturated with 5% nonfat dry milk in T-TBS. Membranes were incubated with primary antibodies overnight at 4 °C and then with peroxidase-conjugated secondary antibody (Jackson Laboratories, Ann Arbor, MI, USA). Antibody detection was performed using ECL (Cyanagen, Italy) and visualized using the ChemiDoc Imaging System (Bio-Rad, Hercules, CA, USA). The intensity of the Western blot bands was quantified using ImageJ. Primary antibodies, diluted according to the manufacturer’s instructions, were as follows: mouse anti-human γ-H2AX and total H2AX, mouse anti-human Ku70, mouse anti-human Rad 51, rabbit anti-human Cyclin D1, rabbit anti-human BAX, rabbit anti-human ATF-4 and mouse anti-human OCT-4 from Santa Cruz Biotechnology (Santa Cruz, CA, USA). The rabbit anti-human Nanog, Snail, vimentin, E-cadherin, RUNX2 and c-FLIP were from Cell Signalling; the rabbit anti-human cleaved Caspase 3 was from Gene Tex (Irvine, CA, USA); and the mouse anti-human BiP was from BD Bioscience. Normalization was performed using HRP-conjugated β-Actin and α-Tubulin (Sigma, St. Louis, MO, USA). All the whole western blot figures can be found in the supplementary materials.

### 2.4. RNA Extraction and RT-qPCR

Total RNA was extracted using Trizol reagent (Invitrogen, Carlsbad, CA, USA). Two µg of total RNA was used for cDNA synthesis. Real-time PCR (qPCR) was carried out using a power up SYBR green master mix (Applied Biosystems, Waltham, MA, USA). Reactions were run in triplicate in three independent experiments. The geometric means of housekeeping genes *β-Actin* or *GAPDH* were used as an internal control to normalize the variability in expression levels. Relative mRNA expression levels were calculated using the ∆∆CT method. Primer sequences are shown in supplementary Table S1. For RNA extraction from floating spheres, sphere-forming cells were obtained by culturing PCa and PCaRR cell lines in anchorage-independent conditions (Corning) in RPMI-medium (PC3 and PC3RR) or in Eagle Minimum Essential Medium (EMEM) (DU-145 and DU-145RR), both supplemented with 10% FCS.

### 2.5. ROS Detection

Cells were detached with 0.25% trypsin/EDTA (Sigma), washed with PBS 0.1% bovine serum albumin (BSA) and incubated with the cell permeant reagent 2′,7′-dichlorofluorescin diacetate (DCFDA, Sigma), following manufacturer’s instructions. DCF fluorescence was detected using flow cytometry analysis, and data were analyzed by employing FlowJo Software.

### 2.6. Migration Assays

The migration assay was performed using transwell membrane filters (8 µm pore size) (Corning). A total of 2 × 10^5^ cells were seeded in the transwell membranes. Cells were seeded in the upper chamber with 1% FCS medium, and 20% FCS medium was added to the bottom chamber. After 24 h, the cells were removed from the top surface of the membrane. The migrating cells adhering to the bottom surface of the membrane were fixed using 4% paraformaldehyde and stained with 600 nM DAPI (Invitrogen). The total number of DAPI-stained nuclei of invading cells was counted under fluorescence microscope by using ImageJ software in seven randomly chosen macroscopic fields/membranes.

### 2.7. Adhesion Assay

Twelve-well plates were coated with 10 μg/mL type I collagen (Sigma) and incubated overnight at 4 °C to allow the collagen to fully polymerize. The day after, 2 × 10^5^ cells were seeded, incubated for 90 min at 37 °C and fixed in 4% paraformaldehyde. Subsequently, cells were stained with crystal violet which was dissolved in 10% acetic acid and then read using a spectrophotometer at 550 nm.

### 2.8. Flow Cytometry

To evaluate the immune phenotype, PC3 and PC3RR cells were detached with 0.25% trypsin EDTA (Sigma), washed with PBS and incubated with bv421-conjugated Integrin alpha-2 antibody (BD Bioscience, San Jose, CA, USA) in PBS/0.1% BSA (Sigma) for 30 min on ice prior to flow cytometric analysis. Propidium iodide (PI) solution (Sigma-Aldrich P4864) was added to exclude dead cells. After washing, cells were assayed using a CyAn ADP flow cytometer (Beckman Coulter, Brea, CA, USA) and data were analyzed using FCS5 Express Software (De Novo Software, Glendale, CA, USA). For cell cycle analysis, PCa and PCaRR cells were fixed with 70% ethanol, washed three times with PBS and stained for 3 h at room temperature with PI, then analyzed using flow cytometry.

To assess the effects of Docetaxel treatment, PC3, PC3RR, DU-145 and DU-145RR cells were seeded 9 × 10^4^/well and 6 × 10^4^/well, respectively, in 12-well plates, then treated with different concentrations (from 10 nM to 100 nM) of Docetaxel (Sigma-Aldrich) for 24 and 48 h. Then, apoptosis/necrosis were assayed by using PI/Annexin Pacific Blue staining (Thermo Fisher Scientific, Rockford, IL, USA) and evaluated using flow cytometry.

### 2.9. Immunofluorescence Analysis

To analyze PC3, PC3RR, DU-145 and DU-145RR cell morphological features, cells were cultured for 48 and 24 h, respectively, in 10% FBS on Ibidi slides (Ibidi, cat. 80826), then fixed in 4% paraformaldehyde in PBS at 4 °C for 10 min. Cells were then permeabilized in PBS 1% BSA/0.1% Triton for 1 h and incubated overnight with mouse anti-vinculin or mouse anti-vimentin antibodies (Santa Cruz, cat. sc-73614 and cat. Sc-6260 Santa Cruz, CA, USA, 1:50 dilution). Samples were washed three times in PBS/BSA/Triton for 30 min and incubated with secondary antibody FITC-conjugated donkey anti-mouse IgG (Jackson Immuno Research, cat. 715-095-150, West Grove, PA, USA, dil. 1:200). TO-PRO3 iodide fluorescent dye 642/661 (1:5000 in PBS, Invitrogen, cat. T3605, Carlsbad, CA, USA) was used for nuclei staining, and rhodamine phalloidin (Invitrogen Molecular Probes Eugene 1:40 dilution) was used for F-actin visualization. Samples were washed three times in PBS/BSA/Triton for 30 min and covered with glycerol–PBS pH 9.5 for confocal microscopy analysis. Immunofluorescence experiments were analyzed using the Zeiss LSM 900 Confocal Microscope Airyscan 2. The images were scanned under a 20× or 40× oil immersion objective. Optical spatial series were performed. Quantitative analysis of vimentin fluorescence intensity was determined on maximum projection of each series and quantified as the SUM (I) of fluorescence/cell. All analyses were performed using Zeiss Confocal software (Zen 3.0 Blue edition).

### 2.10. Mineralization Assay

For the mineralization assay, PC3 and PC3RR cells were cultured in RPMI-1640 plus 10% FBS, 10 mM β-glycerophosphate and 50 µg/mL ascorbic acid to induce mineralization. After five weeks, Von Kossa staining will be performed to evaluate the mineralized area using an image analysis system (NIS Elements BR 4.50.00).

### 2.11. Osteoclast Differentiation

In vitro osteoclast differentiation was performed using Peripheral Blood Mononuclear Cells (PBMC) of 13 healthy donors (HD) in accordance with the rules set by an institutional review board (Policlinico Umberto I, Rome–Ethical Committee, Rif. 3040/16.01.2014 protocol, approval No. 73/14), including the donors’ signatures of informed consent. PBMCs were prepared from EDTA blood samples diluted in PBS solution. Diluted blood was then layered over Ficoll 1.077 g/mL (PANCOLL, PAN Biotec, Germany) and centrifuged at 400× *g* for 30 min. The “buffy coat” was collected and washed twice with PBS. Cells were resuspended in Dulbecco’s modified Eagle medium, containing 50 U/mL of penicillin, 50 mg/mL of streptomycin, 2 mM of L-glutamine and 10% FBS (ThermoScientific). Then, 1 × 10^6^ cells/cm^2^ were plated on a 96-well plate, and after 3 h, cell cultures were rinsed to remove non-adherent cells. PBMCs were cultured in the presence of 50% conditioned medium harvested after 48 h from PC3 and PC3RR cultures and 50% DMEM containing 50 U/mL of penicillin, 50 mg/mL of streptomycin, 2 mM of L-glutamine and 10% FBS. The medium was replaced every 3–4 days. After 14 days, cells were eventually fixed in paraformaldehyde and stained for tartrate-resistant acid phosphatase (TRAcP) and DAPI to evaluate TRAcP-positive multinucleated (>3 nuclei) cells.

### 2.12. Statistical Analysis

Data are presented as the mean ± S.E.M. of results from at least three independent experiments. Student’s *t*-test was used for statistical comparison between means where applicable (two groups). A value of *p* ≤ 0.05 was considered statistically significant.

## 3. Results

### 3.1. Development of PC3RR and DU-145RR Cancer Cell Lines and Characterization of Their Radiosensitivity

**Figure 1:**
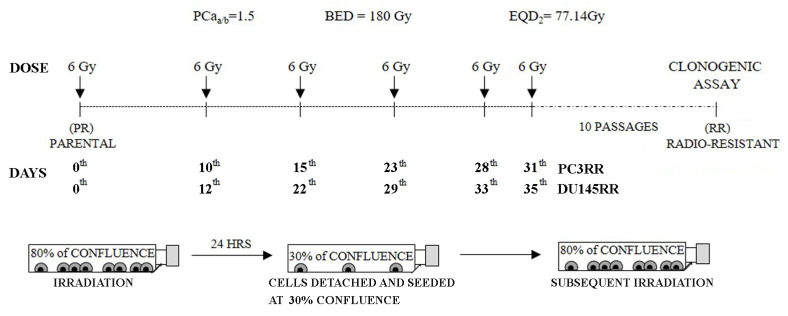
Development of radio-resistant PC3RR and DU-145RR cell lines. Representation of the radiation schedule used and the related radiobiological parameters. Growing PC3 and DU-145 cells at 80% of confluence were irradiated with the dose of 6 Gy. After 24 h, 30% of irradiated cells were re-seeded, and the next irradiation was repeated when a confluence of 80% was reached again. This protocol was applied six times in order to get a final equivalent dose (EQD_2_) that reached conventional fractionation of 77 Gy into daily doses of 2 Gy.

To generate radioresistant (RR) PC3 and DU-145 cell lines, cells were subjected to a ultra-HFRT schedule of six fractions of 6 Gy each. Because cells cultured in vitro are generally more sensitive to treatments than cells cultured in vivo [36], the samples were re-irradiated when they showed a recovery of proliferative potential, as described [27,37] and summarized in Figure 1. Notably, time intervals between subsequent irradiations progressively decreased for both PC3RR and DU-145RR, suggesting the progressive acquisition of resistant phenotypes (Figure 1). We performed comparisons between the radiobiological characterization of PC3RR and DU-145RR cells and that of their parental counterparts (PC3 and DU-145) in order to understand and elucidate the mechanisms underlying cellular radioresistance. To assess the radiosensitivity of PCa and PCaRR cell lines, cells were exposed to increasing doses of ionizing radiations (IR) (2-4-6-8 Gy/min), and clonogenic assay was performed 3 h later. After 11 days of culture, the clones were counted, and the number of colonies in each well was used to calculate the surviving cell fraction, as previously reported [35].

**Figure 2:**
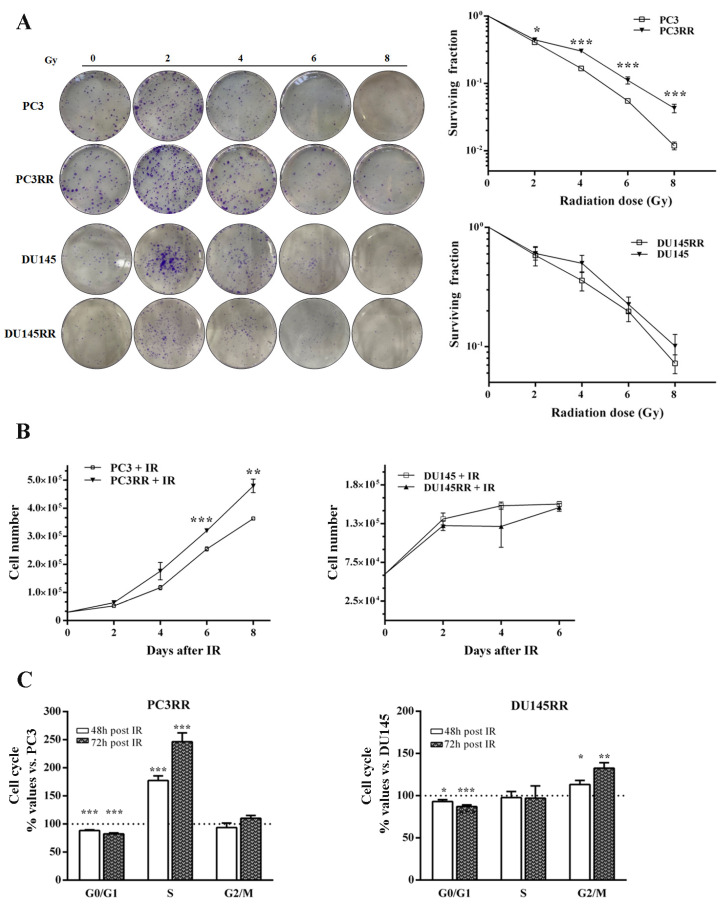
Clonogenic and proliferation assays of parental PCa and PCaRR cell lines. (**A**) Clonogenic assay of the radioresistant cells PC3RR, DU-145RR, parental PC3 and DU-145 cell lines with increasing doses of radiation (2, 4, 6, 8 Gy). Colony was obtained after 11 days of culture post-radiation and stained with crystal violet. The micrographs of the colony formation ability represent plating at 200 and 800 cells/well for the control and IR-treated cells, respectively. The graph shows the surviving fraction after increasing doses of radiation, defined as: n° of colonies formed after IR/n° of cells seeded × plating efficiency (PE). (**B**) Proliferation analysis using cell counting of both PCa and PCaRR cell lines at indicated times after IR at 4 Gy. (**C**) Evaluation of cell cycle distribution at the indicated times after IR at 4 Gy. Data are expressed as the mean ± S.E.M. derived from at least three independent experiments. Statistical significance: * *p* < 0.05; ** *p* < 0.01; *** *p* < 0.001. PC3RR vs. PC3 and DU-145RR vs. DU-145 Student’s unpaired *t*-test.

As shown in Figure 2A, compared to PC3, PC3RR more efficiently formed colonies at each dose tested (2 Gy: PC3RR 0.445 ± 0.009 vs. PC3 0.41 ± 0.015; 4 Gy: PC3RR 0.30 ± 0.01 vs. PC3 0.16 ± 0.008; 6 Gy: PC3RR 0.11 ± 0.01 vs. PC3 0.055 ± 0.002; 8 Gy: PC3RR 0.04 ± 0.006 vs. PC3 0.01 ± 0.001). The ability to recover from IR-induced stress was then assessed by measuring the proliferation rate and cell cycle distribution of PCa and PCaRR cell lines irradiated with 4 Gy, the IR dose chosen for all the subsequent experiments. Compared to PC3, irradiated PC3RR cells more efficiently resumed growth, reaching a statistically significant difference 6 and 8 days after irradiation (Figure 2B, 6 days: PC3RR 320,000 ± 6770 vs. PC3 25,500 ± 7359; 8 days: PC3RR 442,000 ± 41,046 vs. PC3 350,000 ± 13,385). Furthermore, cell cycle analysis shows that the percentage of PC3RR cells in S phase increased 48 and 72 h after IR (Figure 2C). No statistically significant differences were observed between DU-145 and DU-145RR in their ability to form colonies after increasing radiation doses (Figure 2A lower) or to resume proliferation after 4 Gy of IR (Figure 2B). Accordingly, the DU-145RR cell cycle displayed no differences in S phase and in an increased G2/M phase when compared with DU-145 cells (Figure 2C). Altogether, our data suggest that IR selects cells with different behaviours, possibly depending on the metastatic sites from which they originated. 

### 3.2. PC3RR and DU-145RR Cells More Efficiently Repair DNA Damage and Differently Activate an Anti-Apoptotic Pathway after Radiotherapy

**Figure 3:**
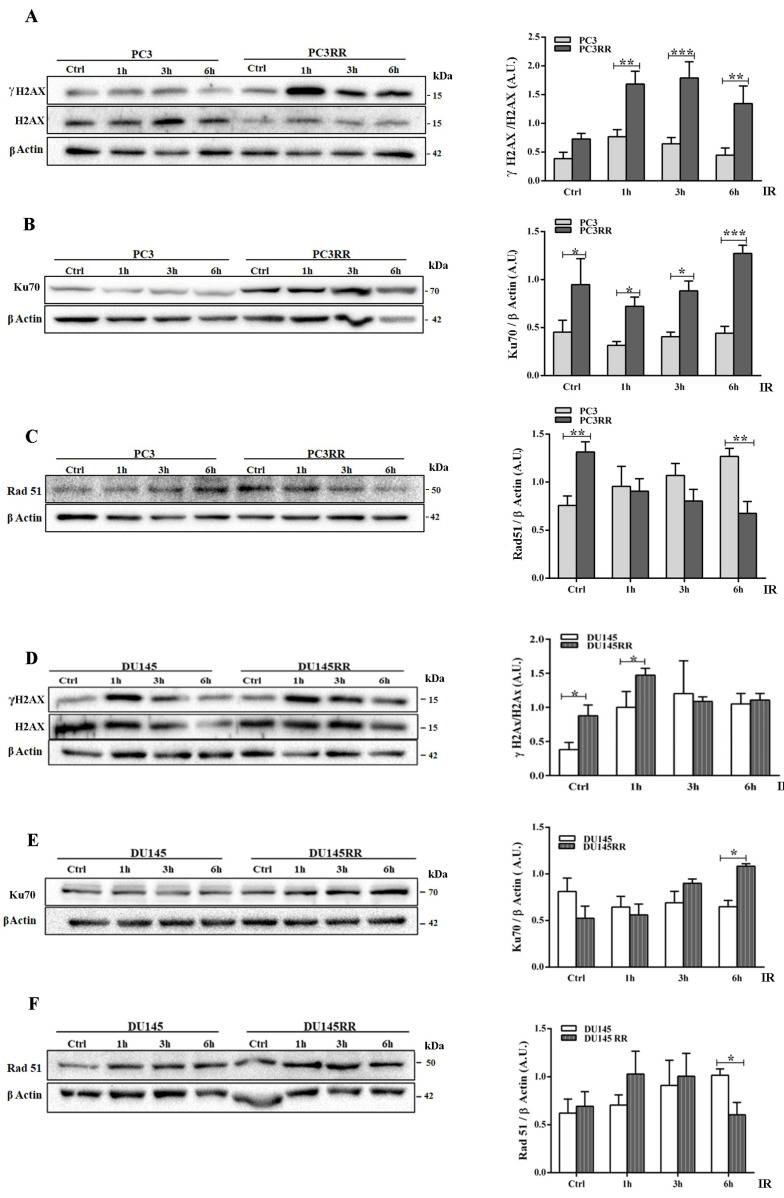
Different DNA repair mechanisms activated by IR in PC3RR and DU-145RR cells compared to their parental counterparts. Cell lysates from unirradiated cells (Ctrl) or cells irradiated with 4 Gy were collected at the indicated times after IR and analysed with Western blot by using specific antibodies against γH2AX/H2AX (**A**,**D**), Ku70 (**B**,**E**) and Rad51 (**C**,**F**). β-Actin or α-Tubulin were used as controls for equal amounts of loaded proteins. Each blot is representative of at least three. The histograms represent the densitometric analysis of Western blot experiments evaluated as arbitrary units (A.U.). Data represent the mean ± S.E.M. derived from at least three independent experiments. Statistical significance: * *p* < 0.05; ** *p* < 0.01; *** *p* < 0.001; PC3RR vs. PC3 and DU-145RR vs. DU-145 Student’s unpaired *t*-test. The whole Western blots were shown in File S1.

IR induces DNA double-strand breaks (DSBs) that trigger a variety of cellular responses, such as the phosphorylation of histone H2AX, to form γH2AX [38], which recruits DNA repair proteins of the non-homologous end joining (NHEJ) and homologous recombination (HR) pathways. The expression levels of γH2AX, a marker of DBSs, and of Ku70 and Rad51, upstream regulators of the NHEJ and HR pathways, respectively, were analyzed by Western blot at 1-3-6 h after IR. As shown in Figure 3A, IR up-regulated γH2AX faster and more persistently in PC3RR cells than in PC3 cells. Accordingly, PC3RR cells stably expressed high levels of Ku70 (Figure 3B) and significantly lower levels of Rad51 protein than PC3 cells at 6 h after IR (Figure 3C). Compared to DU-145, DU-145RR cells showed a higher expression of γH2AX at the basal level, which increased 1h after IR (Figure 3D). Downstream repair molecules Ku70 (Figure 3E) and Rad51 appeared to undergo a late modulation after IR (Figure 3F). These data suggest that PCaRR cells preferentially activate the NHEJ mechanism rather than HR, though to different extents and at different times after IR. Irradiation induces different anti- or pro-survival cellular responses through the expression of apoptotic modulators and proliferation markers correlated with PCa sensitivity to radiotherapy RT [39,40,41]. To elucidate the possible alterations in cell cycle and apoptosis following IR that contribute to radioresistance of PCaRR, we analyzed the expression of key proteins, namely cyclin D1, a mediator of PCa radioresistance [42], pro-apoptotic BAX, anti-apoptotic isoforms of c-FLIP and apoptotic downstream mediator caspase 3.

**Figure 4:**
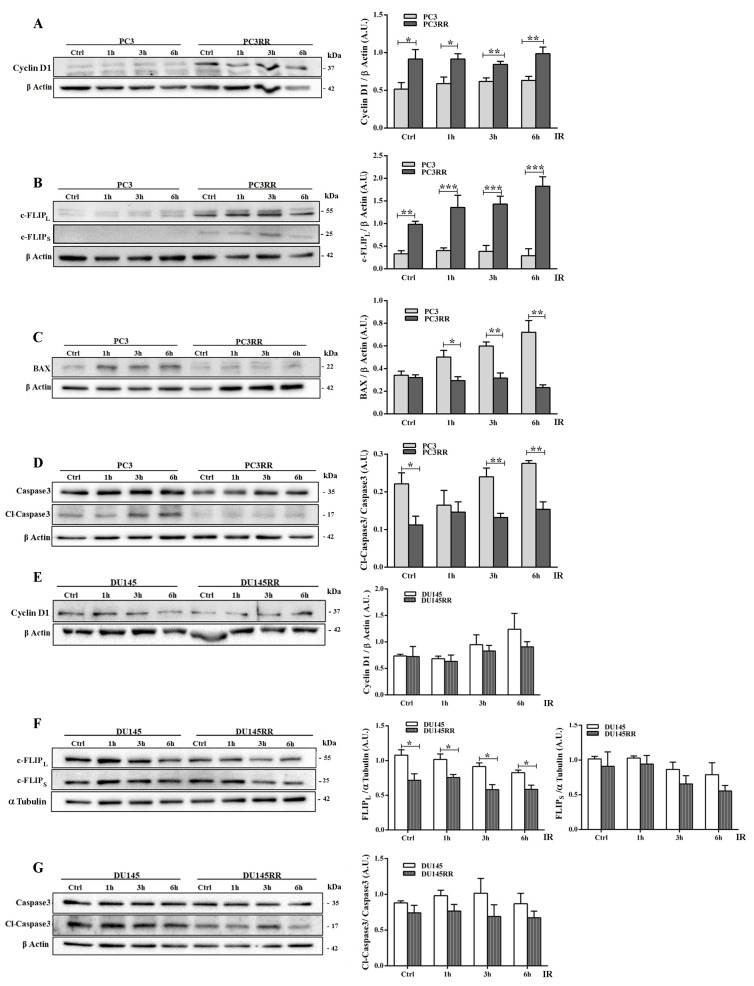
Effect of radiation on proliferation and on apoptotic cell markers in PC3RR and DU-145RR cells compared to their parental counterparts. Cell lysates from untreated or cells treated with 4 Gy were collected at 1, 3 and 6 h after IR. Using Western blots, we analysed the expression of Cyclin D1 (**A**,**E**), c-FLIP_L_ and c-FLIP_S_ (**B**,**F**), BAX (**C**), Caspase 3 and Cl-Caspase 3 (**D**,**G**). β-Actin was used as a control for equal amounts of loaded proteins. Densitometric analysis has been performed on at least three separate Western blots, and the histograms represent the mean ± S.E.M. evaluated as arbitrary units (A.U.). Statistical significance: * *p* <0.05, ** *p* <0.01, *** *p* <0.001; PC3RR vs. PC3 and DU-145RR vs. DU-145 Student’s unpaired *t*-test.

Compared to PC3, PC3RR basally expressed higher levels of cyclin D1, c-FLIP long (c-FLIP_L_) and c-FLIP short (c-FLIP_S_), both efficiently up-regulated by IR (Figure 4A,B). Moreover, PC3RR down-regulated BAX (Figure 4C) and restrained caspase 3 cleavage/activation following IR (Figure 4D). These data indicate that PC3RR cells exhibit more cancer-related survival markers, whereas no difference was detected between DU-145 and DU-145RR cells for proteins involved in anti-apoptotic/proliferative pathways, except for c-FLIP_L_, which was constantly lower in DU-145RR cells (Figure 4E–G). BAX protein was undetectable in either DU-145 or DU-145RR cells.

### 3.3. Surviving Ultra-HFRT Differently Affects Oncophenotype and Migratory Ability of PCa Cells

**Figure 5:**
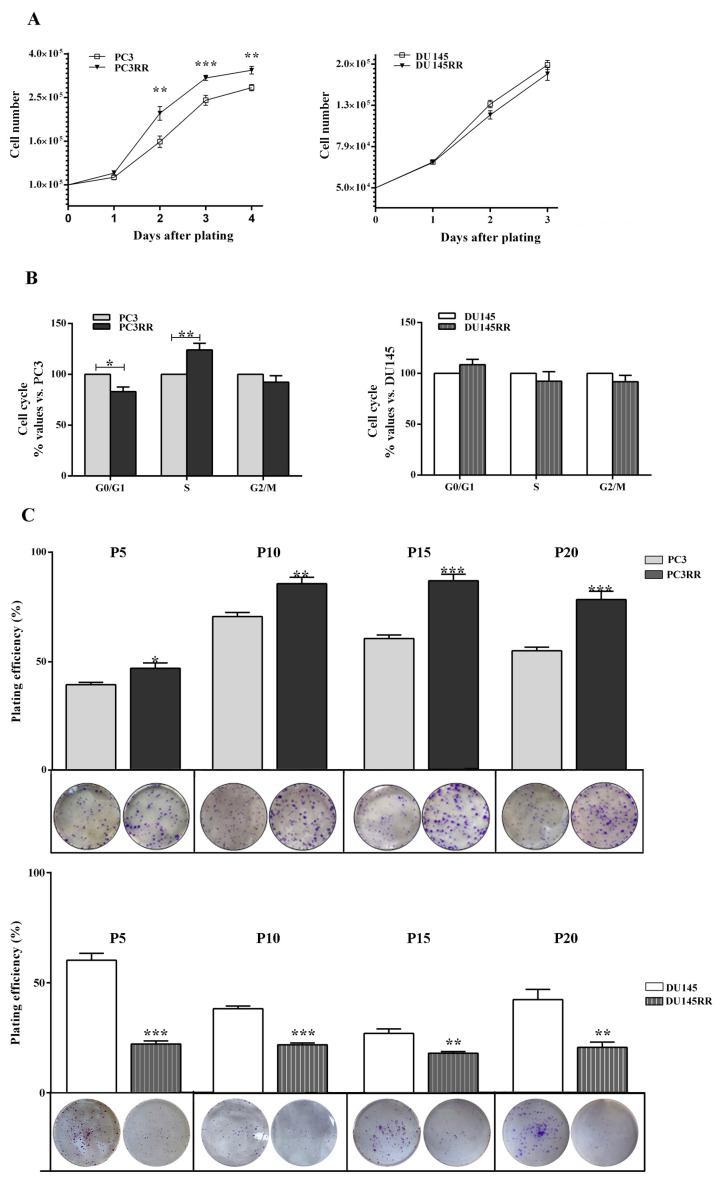
Characterization of proliferation and clonogenic potential of PCaRR cell lines. (**A**) Proliferation and (**B**) cell cycle distribution at 24 h after plating of both cell lines. (**C**) Plating efficiency (PE) is the ratio of the number of colonies to the number of cells seeded x 100. Under each column, the representative micrographs of clones plated at 200 cells/well produced by PC3, PC3RR, DU-145 and DU-145RR cells at different passages (P) from P5 to P20 are shown. Data represent the mean values ± S.E.M. derived from at least three independent experiments. * *p* < 0.05; ** *p* < 0.01; *** *p* < 0.001; PC3RR vs. PC3 and DU-145RR vs. DU-145 Student’s unpaired *t*-test.

The basal proliferation rate, cell cycle distribution, ability to form colonies and cell migration were then assessed in PCaRR and PCa cells. Compared to PC3, PC3RR cells proliferated more efficiently from 2 to 4 days after plating (Figure 5A, 2 days: PC3RR 213,125 ± 15,464 vs. PC3 157,750 ± 9620; 3 days: PC3RR 309,125 ± 8475 vs. PC3 244,375 ± 12,736; 4 days: PC3RR 335,400 ± 13,496 vs. PC3 279,000 ± 9137). No statistically significant differences were observed between DU-145 and DU-145RR (Figure 5A, DU-145RR vs. DU-145). Cell cycle analysis performed at 24 h after plating revealed a significant increase in S phase in PC3RR cells compared to PC3 (Figure 5B, PC3RR vs. PC3), whilst no statistically significant differences were detected between DU-145 and DU-145RR cells (Figure 5B, DU-145RR vs. DU-145). Plating efficiency (PE), measuring the ability to form colonies, was increased in PC3RR (Figure 5C Passage (P) 5: PC3RR 47 ± 2% vs. PC3 39 ± 1%; P10: PC3RR 86 ± 3% vs. PC3 71 ± 2%; P15: PC3RR 87 ± 3% vs. PC3 60 ± 2%; P20: PC3RR 78 ± 4% vs. PC3 55 ± 2%) and decreased in DU-145RR (Figure 5C P5: DU-145RR 22 ± 1% vs. DU-145 60 ± 3%; P10: DU-145RR 38 ± 1% vs. DU-145 21 ± 1%; P15: DU-145RR 18 ± 1% vs. DU-145 27 ± 2%; P20: DU-145RR 21 ± 2% vs. DU-145 42 ± 5%), in both cases stably up to the 20th passage.

**Figure 6:**
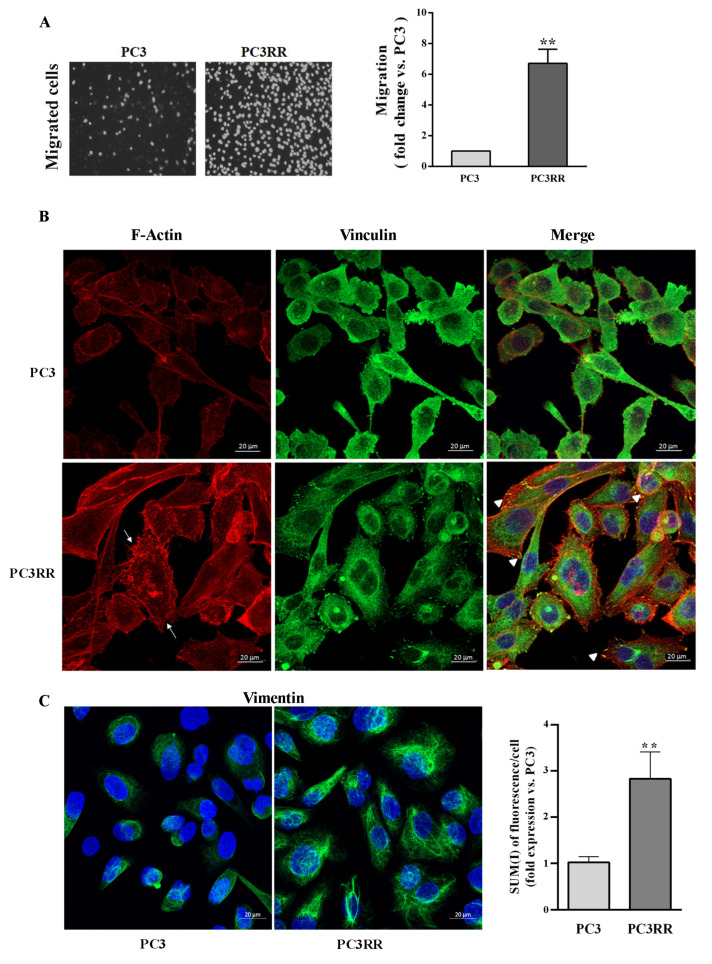
Migration capability and cytoskeleton organization of parental PC3 and PC3RR cells. (**A**) Representative images of trans-well migration 24 h after plating of DAPI-labeled PC3 and PC3RR cells (left). Data are expressed as the fold increase of migration rate relative to PC3 cells (set arbitrarily at one) (right). (**B**) Representative confocal micrographs showing distribution of F-Actin in red (Rhodamine phalloidin), vinculin in green (FITC) and nuclei in blue (TOPRO-3). In PC3RR cells, arrows indicate the lamellipodia and filopodia, and arrowheads indicate Vinculin colocalization with Actin filaments to form focal adhesions (scale bar 20 μm). (**C**) Representative confocal micrographs showing staining of Vimentin in green (FITC) and nuclei in blue (TOPRO-3) (scale bar 20 μm). Graph indicates fluorescence intensity of vimentin, (SUM(I)/cell) performed using Zeiss software. Data are expressed as the mean ± S.E.M. derived from at least three independent experiments. Statistical significance ** *p* < 0.01; PC3RR vs. PC3 Student’s unpaired *t*-test.

The migratory ability and the histological characterization of the cytoskeletal migration-related proteins were investigated by means of trans-well migration in vitro assays and immunofluorescence, respectively. As shown in Figure 6A, migration was enhanced in PC3RR compared to PC3 cells (Figure 6A, PC3RR vs. PC3). In accordance, confocal analysis highlighted a different cytoskeletal organization between PC3 and PC3RR cell lines. Indeed, in PC3RR F-actin forms peripheral cortical bundles, stress fibers, lamellipodia and filopodia structures that are less evident in PC3 cells (Figure 6B). Moreover, vinculin in PC3 cells is mostly cytoplasmatic, while in PC3RR cells, we observed several spots on the cellular membrane that colocalized with F-actin in focal adhesions (Figure 6B). Finally, PC3RR appeared remarkably enriched in vimentin (Figure 6C left) according to the fluorescence intensity analysis, reported as SUM (I)/cell (PC3RR 2.83 ± 0.5 vs. PC3 1± 0.12) (Figure 6C right).

**Figure 7:**
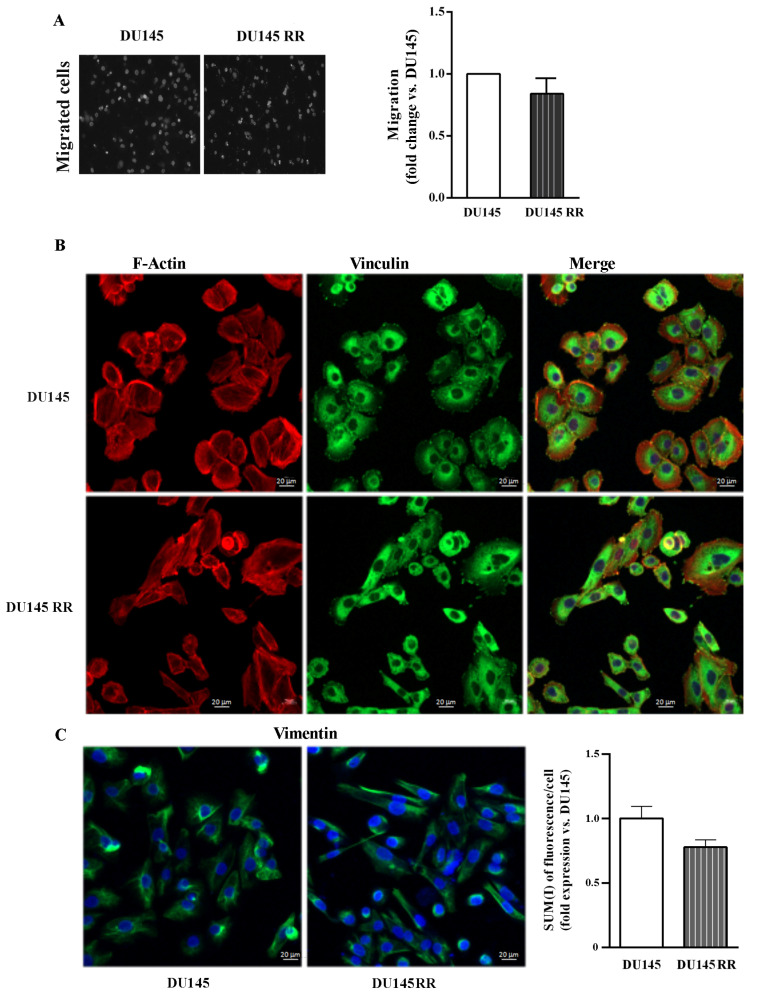
Migration capability and cytoskeleton organization of parental DU-145 and DU-145RR. (**A**) Representative images of trans-well migration 24 h after plating of DAPI-labeled DU-145 and DU-145RR cells (left). Data are expressed as the fold increase of migration rate relative to DU-145 cells (set arbitrarily at one) (right). (**B**) Representative confocal micrographs showing distribution of F-Actin in red (Rhodamine phalloidin), vinculin in green (FITC) and nuclei in blue (TOPRO-3) (scale bar 20 μm). (**C**) Representative confocal micrographs showing staining of vimentin in green (FITC) and nuclei in blue (TOPRO-3) (scale bar 20 μm). Graph indicates fluorescence intensity of vimentin; (SUM(I)/cell) performed using Zeiss software. Data are expressed as the mean ± S.E.M. derived from at least three independent experiments. Statistical significance DU-145RR vs. DU-145 Student’s unpaired *t*-test.

No differences in migratory ability were detected between DU-145 and DU-145RR cells (Figure 7A). The cytoskeletal organization of F-actin and vinculin in DU-145 and DU-145RR cells (Figure 7A) appeared slightly different. Indeed, DU-145RR cells were more scattered and less epithelioid than their parental counterparts. Focal adhesions were observed in both samples, but cell-to-cell-contact vinculin was less evident in DU-145RR than in DU-145 (Figure 7B). No change in either the distribution of vimentin (Figure 7C left) or its expression level, as indicated by SUM (I)/cell (DU-145RR 0.78 ± 0.06 vs. DU-145 1 ± 0.06), was apparent (Figure 7C right). Altogether, this evidence confirms that our sequential irradiation strategy results in increased aggressive behaviour in PC3 cells and much less in DU-145 samples.

### 3.4. PC3RR and DU-145RR Cells Express Mesenchymal Phenotype, Stem Cell Features and a Basal Hyperactivation of Cytoprotective Molecular Mechanisms

**Figure 8:**
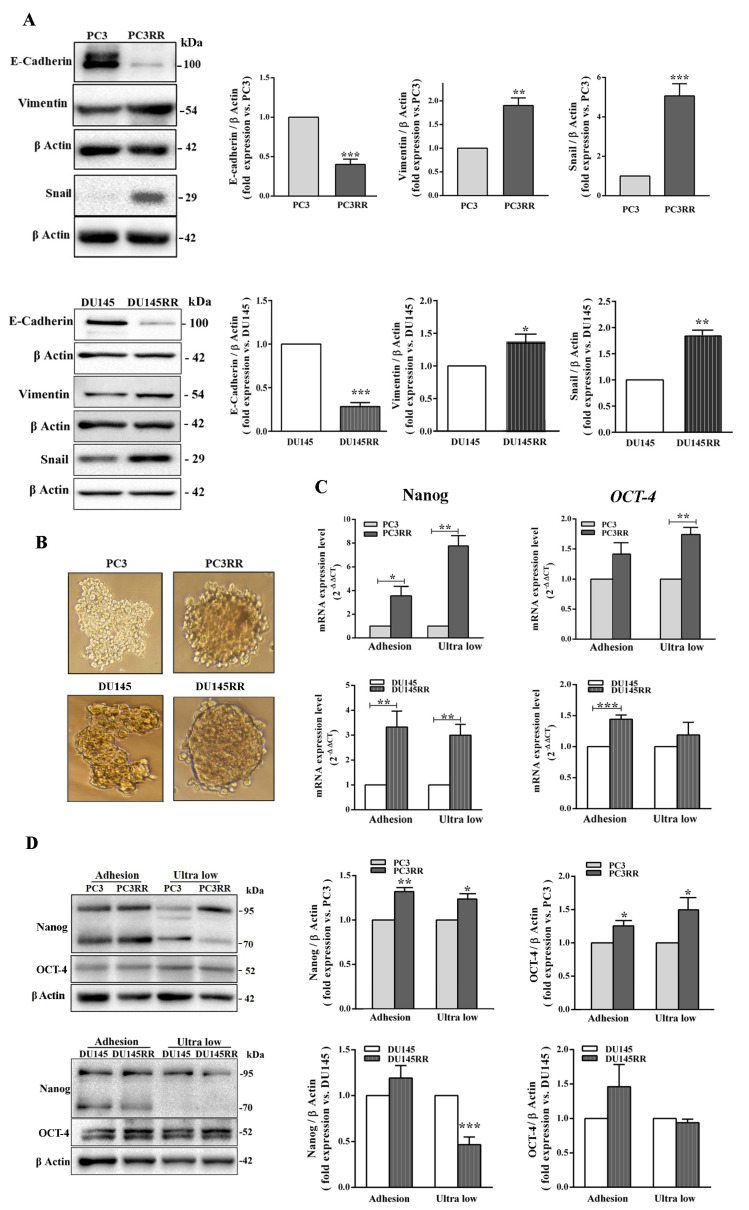
Evaluation of epithelial–mesenchymal transition and stem cell markers in parental PCa and PCaRR cell lines. (**A**) Whole cell extracts were analyzed using Western blot for the indicated epithelial or mesenchymal markers. Each blot is representative of at least three. The histograms represent the densitometric analysis of the Western blot, showing fold expression of each protein normalized with β-Actin (parental PC3 and DU-145 cell value set as one). Data are from at least three separate experiments. (**B**) Phase contrast micrographs of PC3/PC3RR and DU-145/DU-145RR cells cultured in anchorage-independent conditions for 96 and 48 h, respectively. (**C**) The histograms represent the mRNA expression of *OCT-4* and *Nanog* genes assayed by RT-qPCR in PC3/PC3RR and DU-145/DU-145RR cells after 96 and 48 h in adherent or anchorage-independent (ultra-low) conditions, respectively. (**D**) Western blot of PC3/PC3RR and DU-145/DU-145RR cells in the same conditions as in (**C**). Each blot is representative of at least three experiments. The histograms show the densitometric analysis of Western blots, representing the fold expression of the OCT-4 or Nanog (parental PC3 and DU-145 cells value set as one). β-Actin was used as the control for equal amounts of loaded proteins. Data represent the mean ± S.E.M. derived from at least three independent experiments; PC3RR vs. PC3 and DU-145RR vs. DU-145, * *p* < 0.05; ** *p* < 0.01; *** *p* < 0.001, Student’s unpaired *t*-test.

In several cancers, loss of E-cadherin [43], enhanced expression of vimentin [44] and Snail [45] are known to induce EMT that contributes to migration, invasion and radioresistance. Thus, we evaluated the expression of these proteins with a Western blot and observed that PCaRR cells had reduced levels of E-cadherin and increased vimentin and Snail expression compared to their parental counterparts (Figure 8A). It has been reported that cancer recurrence and metastasis may be due to the presence of cancer stem cells (CSCs). We then tested the ability of our cell samples to form three-dimensional spheroid cell clusters (prostaspheres) enriched with CSCs [46]. Compared to PCa cells, when seeded in non-adherent conditions, both PCaRR cell lines formed highly compact prostaspheres (Figure 8B). As expected, PC3RR displayed a stronger expression of the CSC markers *Nanog* and *OCT-4* at mRNA (Figure 8C) and a stronger expression of protein levels (Figure 8D) compared to parental counterparts when cultured in both adherent and non-adherent conditions. Conversely, in DU-145RR cells, despite the increased mRNA of both stem cell markers, we detected no significant upregulation of OCT-4 protein but a marked decrease of Nanog protein, the latter only in non-adherent conditions.

**Figure 9:**
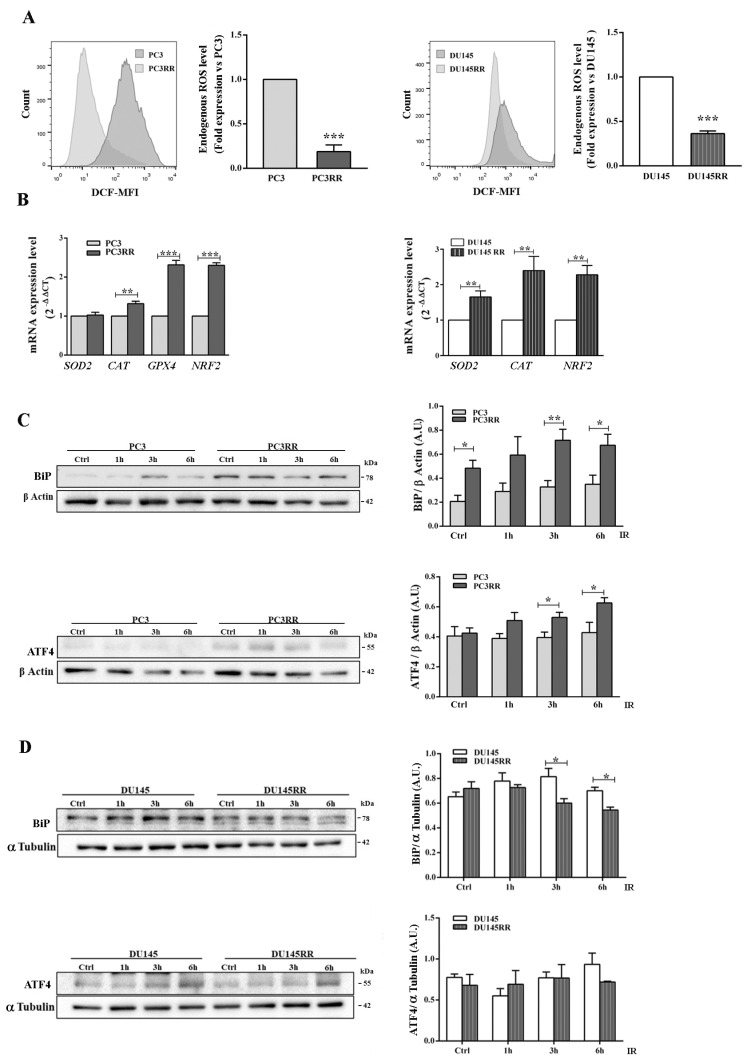
Antioxidant response and UPR activation induced by IR in PC3RR and DU-145RR cells. (**A**) Endogenous ROS levels in parental PCa and PCaRR were measured with flow cytometry using DCFDA dye fluorescence. The graphs represent the fold expression of ROS levels, in which the median of fluorescence of parental PC3 or DU-145 cells is set as one. The values are from three independent experiments. Representative flow cytometry histograms are shown. (**B**) The mRNA levels of the indicated genes were quantified by qRT-PCR, and β-Actin was used as an internal control. To evaluate UPR pathway, Western blot analysis was performed on cell lysates from unirradiated cells (Ctrl) or cells irradiated with 4 Gy and collected 1, 3 and 6 h after IR in PC3/PC3RR (**C**) DU-145/DU-145RR (**D**). β-Actin or α-Tubulin were used as internal controls. Each blot is representative of three experiments. The histograms represent the densitometric analysis of three separate Western blots evaluated as arbitrary units (A.U.). Data represent the mean ± S.E.M. derived from at least three independent experiments. PC3RR vs. PC3 and DU-145RR vs. DU-145; * *p* < 0.05, ** *p* < 0.01, *** *p* < 0.001, Student’s unpaired *t*-test.

Since it is known that CSCs have very low ROS levels in order to potentiate their radioresistance [47], we evaluated the basal ROS levels through flow cytometric analysis and observed that not only PC3RR but also DU-145RR cells had lower ROS levels compared to their respective parental counterparts (Figure 9A). Consistent with this finding, data from RT qPCR experiments showed that the basal expression levels of the anti-oxidant genes *CAT* (catalase) and *NRF-2* were increased in PC3RR and DU-145RR compared to their respective parental cell lines, whereas *GPX4* was higher only in PC3RR and *SOD2* only in DU-145RR cells (Figure 9B).

Another factor that might contribute to adaptive survival signalling in cancer cells during RT is the induction of a ER stress response linked to anti-oxidant properties. The Grp78/BiP chaperone protein, a sensor for misfolded proteins in the ER that triggers the unfolded protein response (UPR), and the UPR-related ATF4 protein [48] were basally upregulated and further increased by IR in PC3RR (Figure 9C). ATF4 was not modulated in DU-145 and DU-145RR, whilst only a late decrease of BiP was observed in DU-145 RR (Figure 9D). This evidence indicates that surviving IR and acquiring radioresistance parallels the enhancement of the EMT program and cancer-stem-like phenotype, as well as an aberrant activation of cytoprotective molecular mechanisms.

### 3.5. PC3RR Cells Present an Upregulated Expression of the Osteoclast Transcription Factors

**Figure 10:**
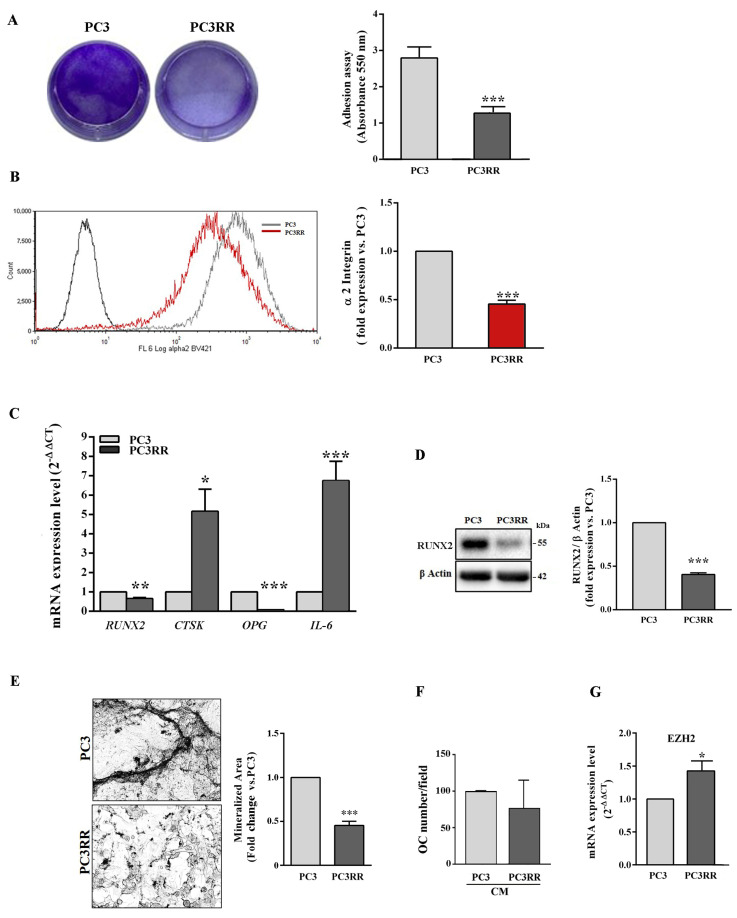
Analysis of genes involved in osteomimicry and functional assay for osteoblast-like activity of PC3 and PC3RR cells. (**A**) Representative wells coated with type I collagen, on which a equal number of PC3 and PC3RR cells were cultured for 90 min and stained with crystal violet. After cell detaching, the absorbance was read using a spectrophotometer. It is shown in the histograms. (**B**) The graph represents the fold expression of the α2 subunit of the integrin receptor, in which the median of fluorescence of parental PC3 cells is set as one (right). Representative flow cytometry histograms are shown (left). (**C**) The histograms represent RT-qPCR analysis of genes involved in osteoblast and osteoclast differentiation and function. (**D**) Representative blot of RUNX2 (left). The graph shows fold increase of RUNX2/β-Actin ratio of PC3RR cells vs.PC3 (right). (**E**) Representative image of PC3 and PC3RR cells ability to release mineralized nodules, revealed by Von Kossa staining (left). The graph represents the fold of the mineralized area with parental PC3 value set as one (right). (**F**) Effect of conditioned medium from PC3 and PC3RR cells on the osteoclast differentiation of PBMCs isolated from 13 healthy donors. PBMCs were cultured in the presence of 50% conditioned medium (CM) obtained from PC3 and PC3RR cultures. After 14 days, the number of multinucleated TRAcP positive cells was evaluated. The results are expressed as a percentage vs. osteoclast number obtained from the differentiation of PBMCs in the presence of CM from PC3. (**G**) RT-qPCR analysis of *EZH2* expression levels in PC3/PC3RR. All data represent the mean ± S.E.M. derived from at least three independent experiments. Statistical analysis * *p* < 0.05; ** *p* <0.01; *** *p* < 0.001; PC3RR vs. PC3.

The ability to adhere to type I collagen, the most abundant component of organic extracellular matrix in bone tissue, and the expression of the α2 subunit of type I collagen receptor α2β1 were assessed on bone-metastasis-derived PC3 and PC3RR. As shown in Figure 10A, compared to PC3, PC3RR cells adhered to type I collagen (Figure 9A) and down-regulated the expression of α2 subunit less efficiently(Figure 10B). The expression of the master transcription factor of osteoblast differentiation, runt-related transcription factor (RUNX2), osteoclast function-related cathepsin K (CTSK), osteoclastogenesis inhibitory factor osteoprotegerin (OPG) and interleukin-6 (IL-6) was investigated using RT-qPCR. Compared to PC3, PC3RR cells expressed lower levels of *RUNX2* and *OPG* mRNA and higher of *CTSK* and *IL-6* (Figure 10C). The lower expression of RUNX2 in PC3RR cells was also confirmed using Western blot (Figure 10D). A mineralization assay shows that PC3RR released mineralized nodules less efficiently, as revealed by Von Kossa staining (Figure 10E). To investigate the ability of tumour cells to support osteoclastogenesis, we treated peripheral blood mononuclear cells (PBMC) isolated from healthy donors with a conditioned medium (CM) isolated from PC3 and PC3RR cells. As shown in Figure 10F, no statistically significant differences were detected in the ability to regulate osteoclast differentiation between PC3 and PC3RR cells. These results suggest that PC3RR cells are likely more prone to leave the bone, consistent with their mesenchymal features. The result is that they promote osteolytic lesions more efficiently compared to PC3 cells. Moreover, enhancer of zeste homologue 2 (EZH2) has been recently implicated as a master regulator of secondary metastases from bone lesions [49]. Thus, we assessed *EZH2* expression level using RT-qPCR, and Figure 10G shows a higher *EZH2* expression in PC3RR cells compared to parental cells.

### 3.6. PC3RR Cells Are More Susceptible to Docetaxel Treatment

**Figure 11:**
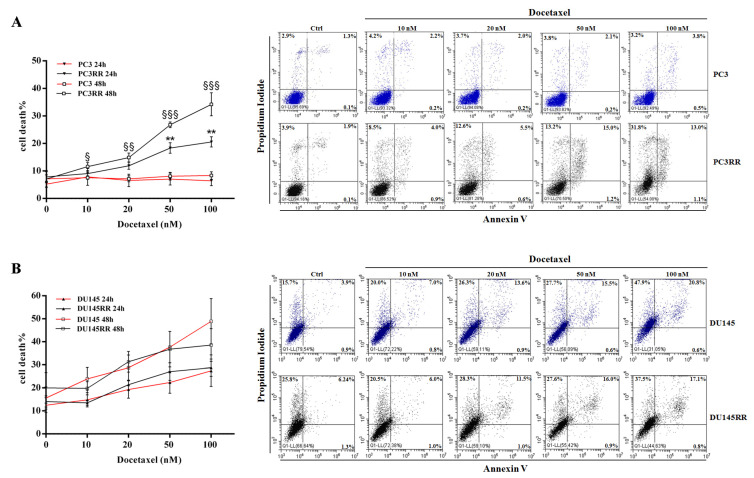
Effect of Docetaxel treatment on the viability of PC3/PC3RR cells and DU-145/DU-145RR cells.(**A**) Representative dot plot of flow cytometry analysis for Annexin-V/Propidium Iodide staining in PC3/PC3RR ((**A**), right) and DU-145/DU-145RR cells ((**B**), right) to evaluate percentage of dead cells after treatment with different concentrations of Docetaxel at 48 h. For each dot plot, necrotic cells are localised in the upper left panel, whereas late and early apoptotic cells are in the upper and down right panels, respectively. The graphs show the percentage of cell death at 24 and 48 h after various doses of Docetaxel for PC3/PC3RR ((**A**), left) and DU-145/DU-145RR cells ((**B**), left).Data represent the mean of cell death percentage ± S.E.M. derived from at least three independent experiments. Statistical analysis ** *p* < 0.01, PC3/DU-145 24 h vs. PC3RR/DU-145RR 24 h, § *p* < 0.05, §§ *p* < 0.01, §§§ *p* < 0.001, PC3/DU-145 48 h vs. PC3RR/DU-145RR 48 h.

Metastatic patients are usually treated with hormonal therapy, but when cancer cells become unresponsive, other strategies must be used, such as chemo or radiation therapy, sometimes with palliative intent [50]. Therefore, we examined sensitivity patterns to Docetaxel treatment in the parental cell lines and in the corresponding radiation-resistant ones. To this aim, we treated parental PCa and PCaRR cells with increasing doses of Docetaxel for 24 and 48 h, and then we performed flow cytometry analysis by Annexin-V/PI staining to evaluate the apoptotic/necrotic cell percentage. As shown in Figure 11, Docetaxel treatment of PC3RR induces a significant increase in the percentage of cell death at 50–100 nM after 24 h, and at all doses after 48 h, compared with parental PC3 (Figure 11A). The percentage of necrotic and early/late apoptotic cells, at all Docetaxel doses and at all times tested, is detailed in supplementary Table S2. Conversely, a similar rate of cell death was observed in DU-145 and DU-145RR at all doses tested (Figure 11B).

## 4. Discussion

Understanding the molecular mechanisms driving radioresistance is crucial to improving the therapeutic efficacy of HFRT in order to identify personalized RT-based strategies. In this study, we present for the first time a new model of two prostate cancer cell lines, PC3 and DU-145, either in the native state or after radiation treatment with ultra-HFRT, which is at present one of the clinical strategies to cure prostate cancer patients [33]. In our study, the cells were re-irradiated in vitro as soon as they showed signs of resistance, such as recovery of proliferation. PC3RR and DU-145RR cell lines were selected after a complete course of ultra-HFRT that, using a larger dose per fraction, we supposed would exalt the molecular mechanisms used by PCa to survive high dose of RT. Notably, unlike clinical practice—in which any single fraction of 6 Gy is delivered weekly—herein, considering the higher susceptibility of in vitro cells to any type of stress, including RT, we re-irradiated as soon as the cells showed signs of recovery in terms of proliferation. The progressive reduction in the time-intervals between fractions suggested that PCa cells were acquiring radioresistance [27]. However, while this hypothesis was confirmed for PC3RR cells, compared to the parental counterpart, irradiated DU-145RR did not show any statistically significant increase in its ability to form colonies. However, it has been shown that cancer cells selected for resistance to RT can form colonies less efficiently when further irradiated, and that other non-clonogenic assays should be performed to assess the acquired radioresistance for these cells [51]. The ability of cancer cells to repair damaged DNA, redistribute cell cycle phases, repopulate and reoxygenate after IR represent the “4Rs” of radiobiology, suitable for stratifying cancer patients into responders and non-responders to RT [52]. Thus, we decided to explore the “4Rs” in order to identify the molecular mechanisms that potentially sustain PCa survival of ultra-HFRT. RT kills cancer cells mainly by inducing the accumulation of DSBs and the consequent activation of several cell death programs, including apoptosis [38]. DSBs can be induced directly by IR or indirectly, through water radiolysis and the consequent accumulation of intracellular ROS [53]. Thus, the survival of cancer cells after RT mainly depends on their ability to activate DNA damage repair and ROS detoxifying pathways [12]. In this respect, PC3 cells have been previously shown in an in vitro study to be sensitive to ROS production and to be rescued by drug-induced apoptosis by reducing intracellular ROS generation and pro-inflammatory markers [54]. It has been shown that repeated DNA damage caused by subsequent irradiation can generate genomic instability responsible for increased susceptibility to RT [55]: unrepaired DSBs induce permanent cell growth arrest or death [41]. γ-H2AX levels, a biomarker of damaged DNA, were basally higher only in DU-145RR cells compared to their parental counterparts, and was more significantly induced by RT in both PCaRR, transiently in DU-145RR and stably in PC3RR. Since high basal levels of γ-H2AX have been related to radiosensitivity [56], we suppose that ultra-HFRT has increased genomic instability in DU-145RR, favouring the accumulation of mutations that will be further investigated. γH2AX binds to the chromatin surrounding a DSB, quickly recruiting DNA repair factors, which form multiprotein complexes around it [57]. The main pathways responsible for DNA repair are represented by the NHEJ and HR pathways, which are mainly regulated by Ku70/80 and RAD51 proteins, respectively. NHEJ is the predominant repair pathway in human cells, preventing genomic instability through the repair of DSBs [58]. PCaRR showed an increased ability to repair DNA damage by upregulating the expression of Ku70, though to a different extent in PC3RR and DU-145RR cells. The preferential activation of the NHEJ pathway in both PCaRR was confirmed by downregulation of RAD51 after IR. Ku proteins have been found to be overexpressed in several cancer types, and the loss of the NHEJ factors, including Ku70, has been shown to lead to genomic instability, as well as an increased sensitivity of cells to genotoxic agents, including IR [59]. We therefore hypothesize that higher basal expression of γH2AX and the late activation of Ku70 induced by ultra-HFRT in DU-145RR cells could contribute to their lower clonogenicity after IR.

RT-induced cell cycle arrest can predispose to a greater or lesser sensitivity to further radiation, with the G2/M and G1/S phase of the cell cycle known to be the most and least radioresponsive, respectively. The redistribution indicates the different ability of cells to escape from RT-induced cell cycle arrest. Thus, cancer cells that leave G2/M or persist in G1/S cell cycle arrest more easily survive subsequent irradiations [37]. In accordance with their higher radioresistance, PC3RR recovers the S phase more efficiently than PC3, whilst DU-145RR persists in the G2/M phase, suggesting that the latter radioresistant cell line may include a partially radiosensitive subpopulation. The increased S phase in PC3RR cells is an indication of tumour repopulation after an efficient activation of DNA repair mechanisms defined as the ability of tumour cells to continue proliferating after IR, and is also known to be responsible for the failure of IR. In accordance with the higher ability of PC3RR to repopulate, we found that these cells, compared to their parental counterparts, more efficiently upregulated the expression of the cell cycle promoter cyclin D1 [42], anti-apoptotic c-FLIP_L_ and c-FLIP_S_ isoforms [60] while downregulating the expression of pro-apoptotic BAX protein [61]. Notably, cyclin D1 has been shown to be required for cell cycle progression in G1 [62] and to mediate resistance to apoptosis [63] and promote PCa radioresistance by sustaining DSB repair [42]. These results suggest that PC3RR could activate a survival strategy after radiation treatment, whereas DU-145RR did not modify life/death protein expression and cell cycle distribution compared to its parental counterpart.

Notably, increasing evidence indicates cancer stem cells (CSCs), a subpopulation within tumours with an unlimited potential of cell division, to be the main culprits of repopulation [64]. Accordingly, both PCaRR cell lines express a more CSC-like phenotype, as indicated by their increased ability to form tumour spheres enriched in PCa cells expressing stemness-related markers, in accordance with what has already been described in other RR cell lines [27,29,37,65]. Moreover, various cancer types, including PCa, display enhanced DNA repair capabilities, and CSCs have been shown to have altered DNA damage responses and repair pathways [66,67,68,69] that lead to the failure of tumour therapy, including RT. Particularly, NHEJ proteins, herein found to be upregulated in PCaRR, have been considered important for increased radioresistance of CSCs [70,71]. Thus, ultra-HFRT could enrich the stem-like phenotype, exalting the radioresistance-related properties of CSCs in PCa. Pre-clinical evidence suggests that RT can promote selection and enrichment in CSCs [53,72] including in PCa, improving the pro-metastatic phenotype [24]. The enrichment of CSCs could depend on the intrinsic higher radioresistance of CSCs compared to non-CSCs, which could promote a relative increase in CSC number. However, increasing reports support the idea that non-CSCs exhibit a remarkable degree of plasticity that allows them to re-acquire CSC traits in the context of RT. To verify whether non-CSC plasticity could be responsible for CSCs’ enrichment, in our samples, we investigated the epithelial–mesenchymal transition (EMT), known to be closely linked to CSCs [73] and to improve the migratory and invasive traits [53,74,75] of cancer cells, including PCa [76]. In accordance with our hypothesis, we found that both PCaRR expressed higher levels of EMT markers compared to their respective parental counterparts, and PC3RR cells also displayed an increased migration ability. This indicates that the use of a higher dose per fraction can induce tumour plasticity by activating EMT, and that targeting EMT during RT could be another valid strategy to counteract acquired radioresistance, as recently reported [77]. Moreover, the radioresistance of CSCs has been related to lower levels of intracellular ROS, associated with elevated free-radical scavengers [47] that further permit to more efficient neutralization of ROS. In line with this evidence, we found that, compared to their parental cell lines, both PC3RR and DU-145RR had significantly lower levels of basal ROS and a higher expression level of genes associated with ROS scavenging, consistent with radioresistant lung cancer cells [78] and radioresistant triple negative breast cancer cells [79]. Increase of EMT markers and lower ROS level in DU-145RR cells are not in line with CSC protein expression. These results could suggest that mesenchymal phenotype and low ROS are not sufficient to induce the enrichment of stem features in PCa metastases.

During bone invasion, tumour cells produce several transcription factors involved in osteoblast and osteoclast differentiation, a process known as osteomimicry [80]. Notably, we observed that, compared to PC3, PC3RR cells displayed a reduction in osteoblast features and mineralization ability, but increased expression of the osteoclast marker cathepsin K involved in bone remodelling, characteristic of osteolytic lesions, suggesting a more aggressive invasive phenotype. The presence of cathepsin K in the sera of PCa patients has been associated with tumour aggressiveness and is regarded as a contribution to bone resorption [81]. Moreover, our experiments revealed that PC3RR cells secrete factors, such as IL-6, capable of stimulating the bone erosion that creates the physical space in which tumour cells can grow and reach a critical mass. Therefore, it has been hypothesized that the aggressiveness of these cells is underpinned by interplay between tumour and bone microenvironments that promote PC3RR cell invasiveness through micrometastases in other sites [49]. Strikingly, more than two-thirds of patients with bone lesions develop metastasis in secondary organs. This phenomenon seems to be related to the ability of the bone microenvironment to induce phenotypic changes in cancer cells, such as generation or selection of CSCs [49]. In accordance with this hypothesis of ours, PC3RR expresses higher EZH2, a factor recently shown to be involved in secondary metastatic spread from the microenvironment, rather than in tumour growth [49].

Interestingly, as a point of convergence for all the evidence described so far, we found that PC3RR upregulated the expression of unfolded protein response (UPR) regulator GRP78/BiP, ATF4 and PERK, basally and after IR. GRP78/BiP is a major endoplasmic reticulum (ER) chaperone protein critical for protein quality control that acts by regulating several downstream targets, including ATF4 and PERK [48]. GRP78/BiP promotes radioresistance of several cancer types [82], supporting DNA repair [83], anti-oxidant activity [84], metastases [85] and cancer stemness [86]. On the contrary, DU-145RR cells did not upregulate either UPR response after IR or basal migratory ability, suggesting that different PCa cell lines that survived ultra-HFRT do not display the same grade of aggressive phenotype.

Finally, we found that acquiring radioresistance parallels a higher sensitivity to Docetaxel in PC3RR cells, but not in DU-145RR cells. The immediate clinical implication of this novel finding could be that prostate cancer patients with disease progression after radiotherapy, especially when developing distant metastasis, could be particularly sensitive to upfront chemotherapy, harbouring subsets of cells with increased drug-sensitivity. However, it remains to be investigated why PC3RR cells and DU-145RR cells show different sensitivity to taxanes. Interestingly, it has recently been that there is biological heterogeneity of the radiation-surviving cell subpopulations in PCa, including their phenotypic plasticity, stem-like cell properties and tumorigenic abilities [87]. On the basis of these findings, we can speculate that radiation-resistant prostate cancer cells may include various subpopulations of cells displaying different traits of chemo/radiosensitivity or resistance, such as mesenchymal markers, invasion/migration ability, low basal ROS levels, CSC markers and ER-stress defence machinery.

## 5. Conclusions

The data presented in this study seem to confirm that increasing the dose per fraction in clinical practice by using HFRT or ultra-HFRT is insufficient to overcome the intrinsic radioresistance of PCa and to prevent the acquisition of a more aggressive pro-metastatic phenotype. A patient-tailored approach based on molecular characteristics of specific prostate cancer cellular subtypes seems to be a more beneficial strategy, also considering target radiosensitizing therapies and the upfront use of chemotherapy after radiation-induced chemo-sensitization of cancer cells. All the molecular complex mechanisms responsible for radioresistance and chemosensitization in prostate cancer need to be further investigated in the future. One possible promising way to do this is to use isogenic models of induced radioresistance, such as the one reported and analysed in the present study.

## Data Availability

The data presented in this study are available on request from the corresponding author.

## Supplementary Materials

The following supporting information can be downloaded at: https://www.mdpi.com/article/10.3390/cancers14225504/s1, File S1: the whole western blots; Table S1: primer sequences; Table S2: Percentages of necrotic and early/late apoptotic after Docetaxel treatment. All the whole western blot figures can be found in the supplementary materials.
