# Supplementary material for: Radioresistance Mechanisms in Prostate Cancer Cell Lines Surviving Ultra-Hypo-Fractionated EBRT: Implications and Possible Clinical Applications
Source: Cancers (Basel). 2022 Nov 9;14(22):5504. doi: 10.3390/cancers14225504 (PMC9688510; doi:10.3390/cancers14225504)
Supplement: Supplementary file 1 [file cancers-14-05504-s001.zip › cancers-2010478 the whole western blot.pdf]

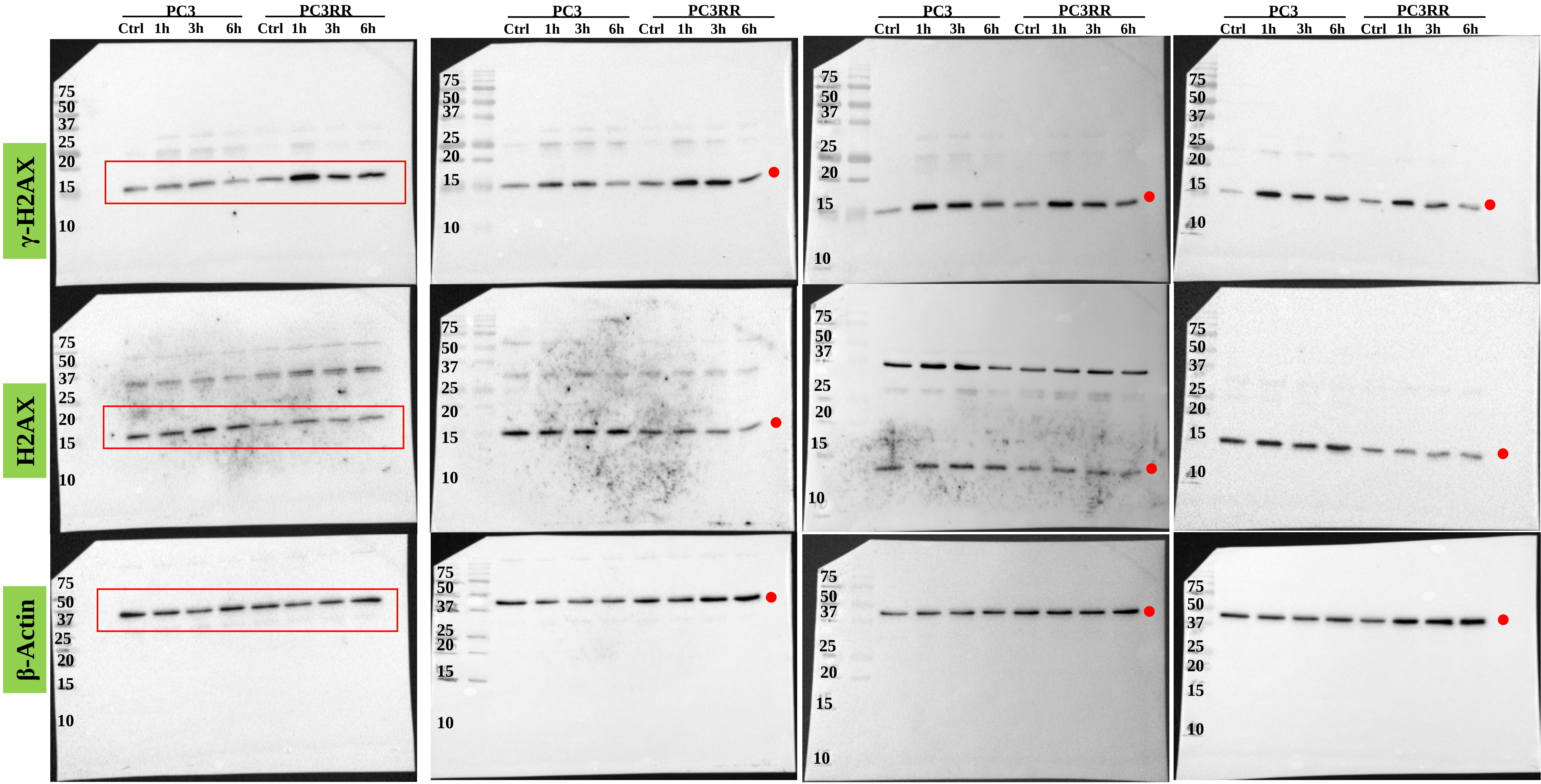

Figure. S3A

The **RED BOXES** indicates the lanes of the WB selected for the figures included in the paper

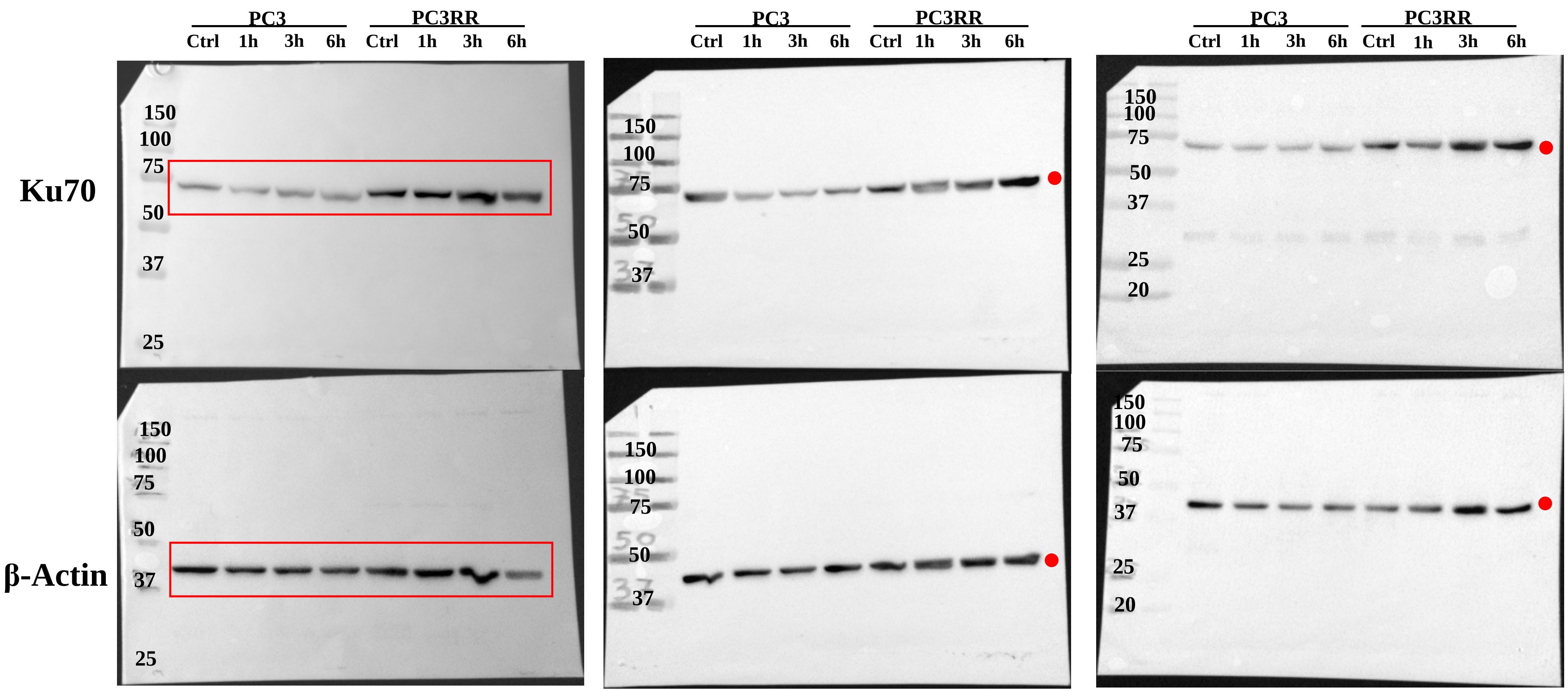
PC3
PC3RR
Ctrl 1h 3h 6h Ctrl 1h 3h 6h
Ku70
β-Actin
150
100
75
50
37
25
20

Figure. S3B

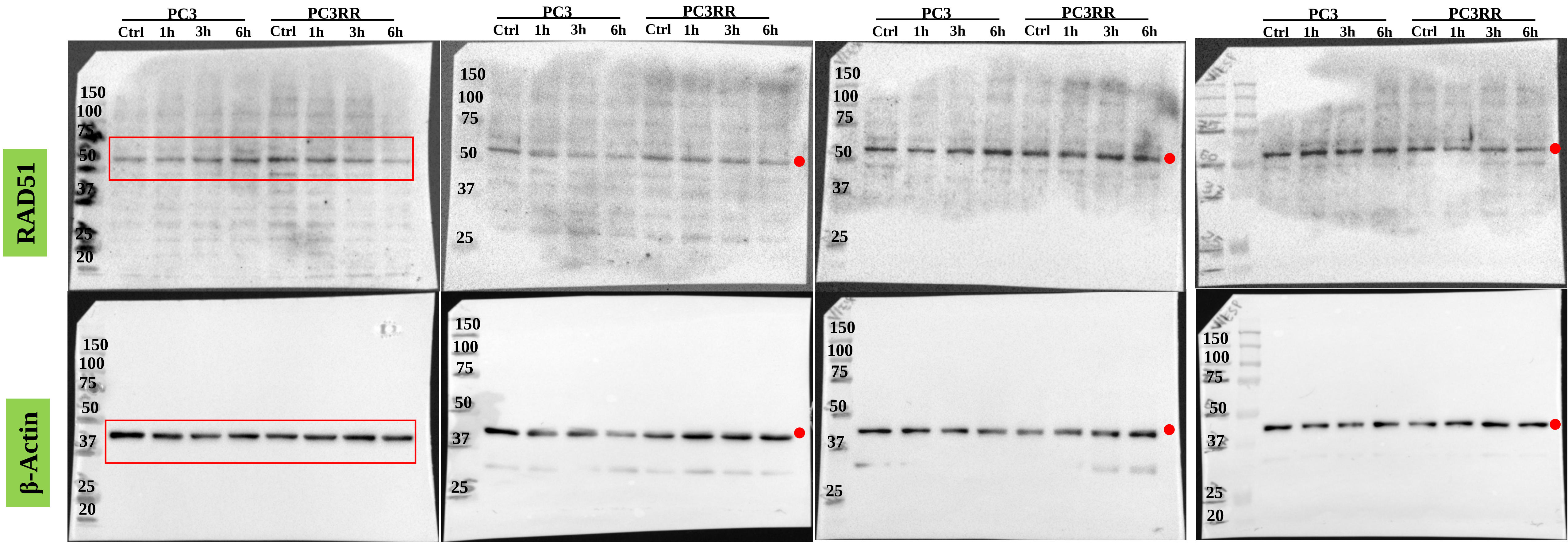
PC3
PC3RR
Ctrl 1h 3h 6h Ctrl 1h 3h 6h
RAD51
β-Actin
150
100
75
50
37
25
20

Figure. S3C

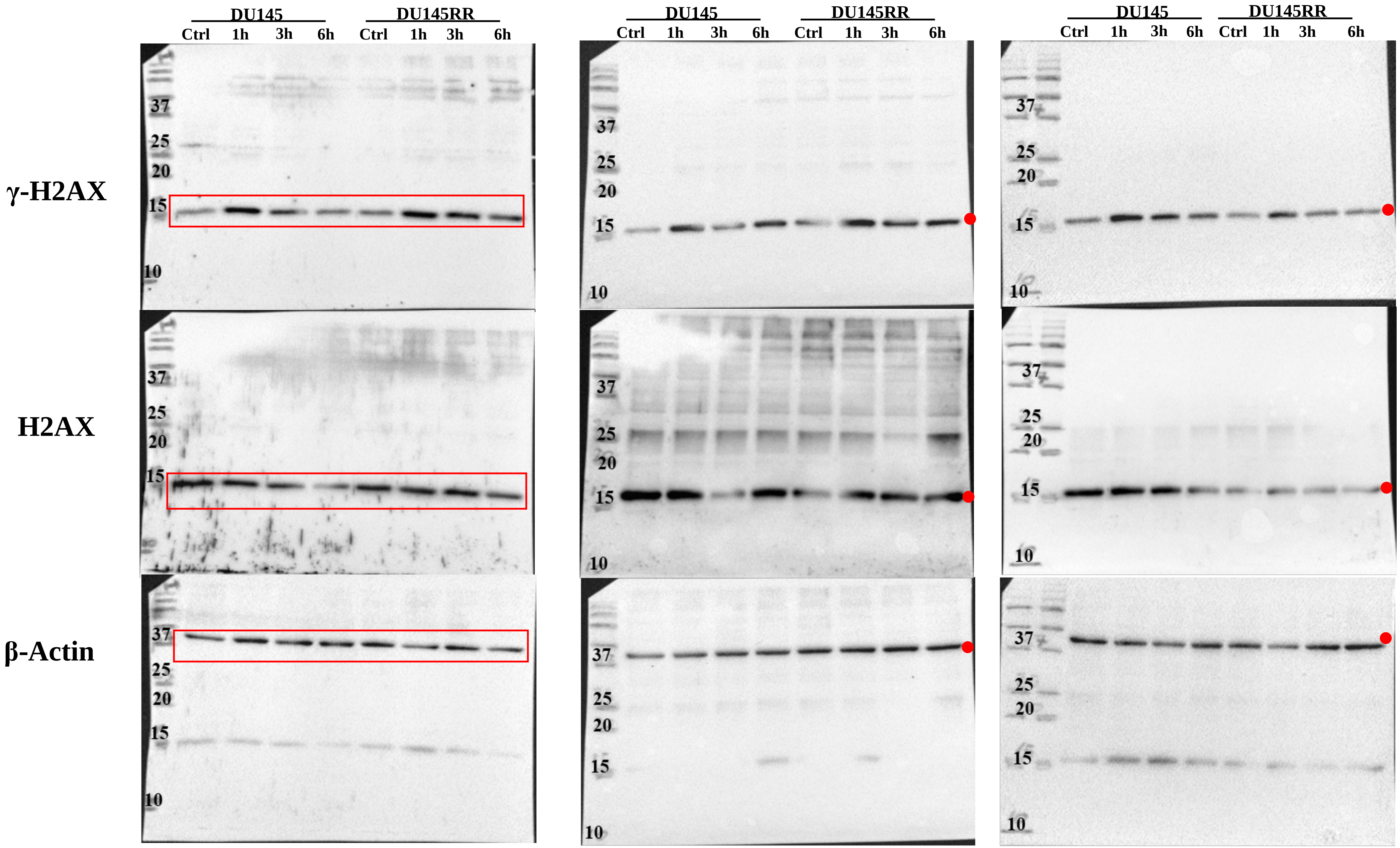

Figure. S3D

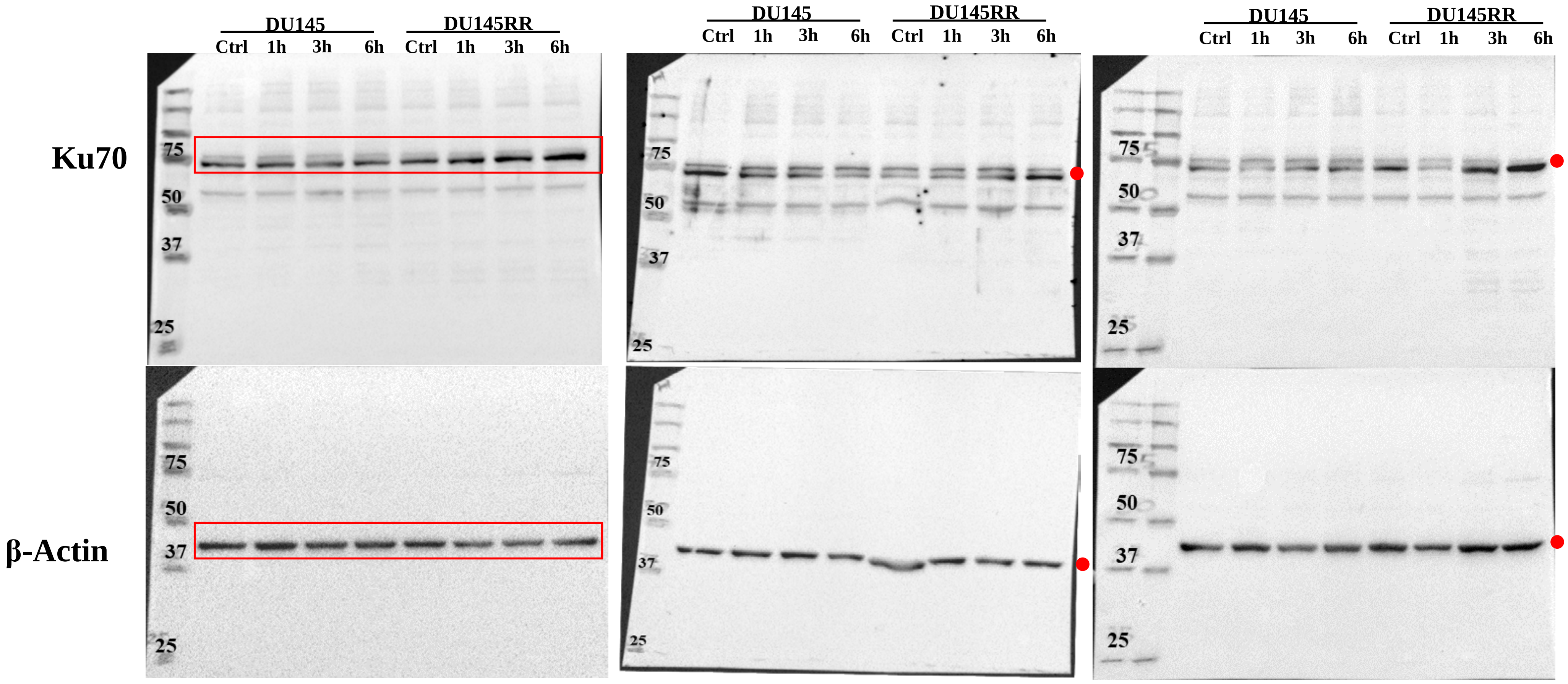

DU145
DU145RR
Ctrl 1h 3h 6h Ctrl 1h 3h 6h
Ku70
β-Actin
75
50
37
25

Figure. S3E

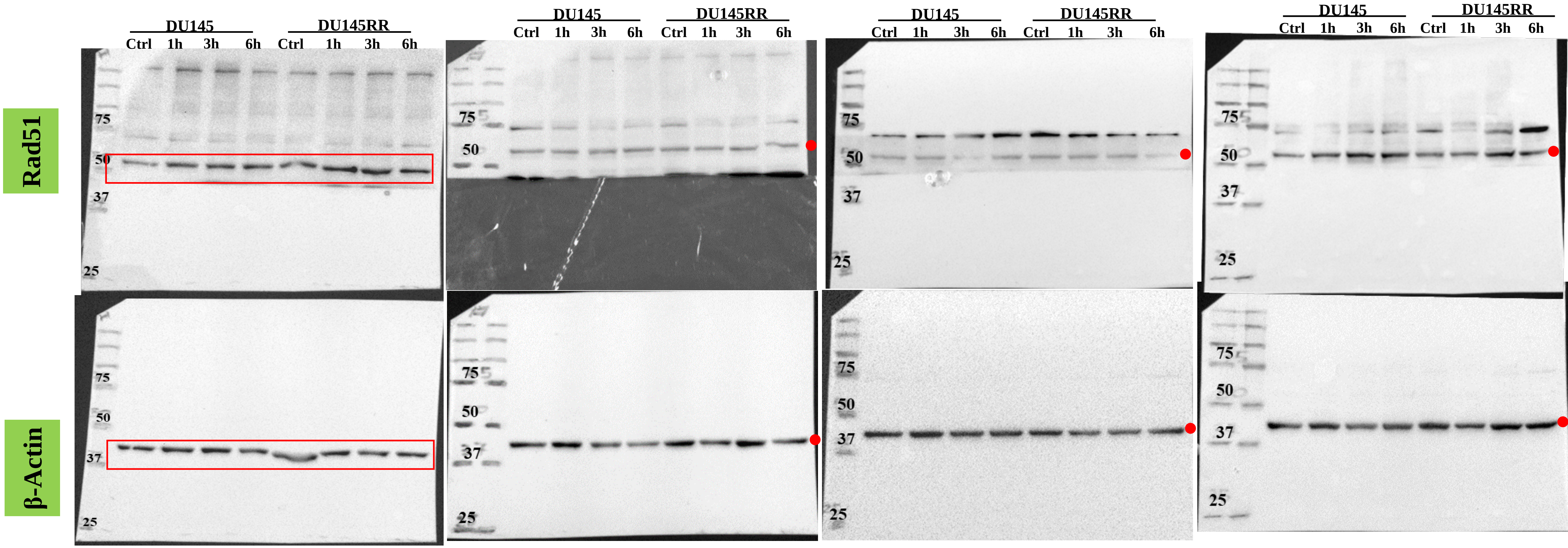

Figure. S3F

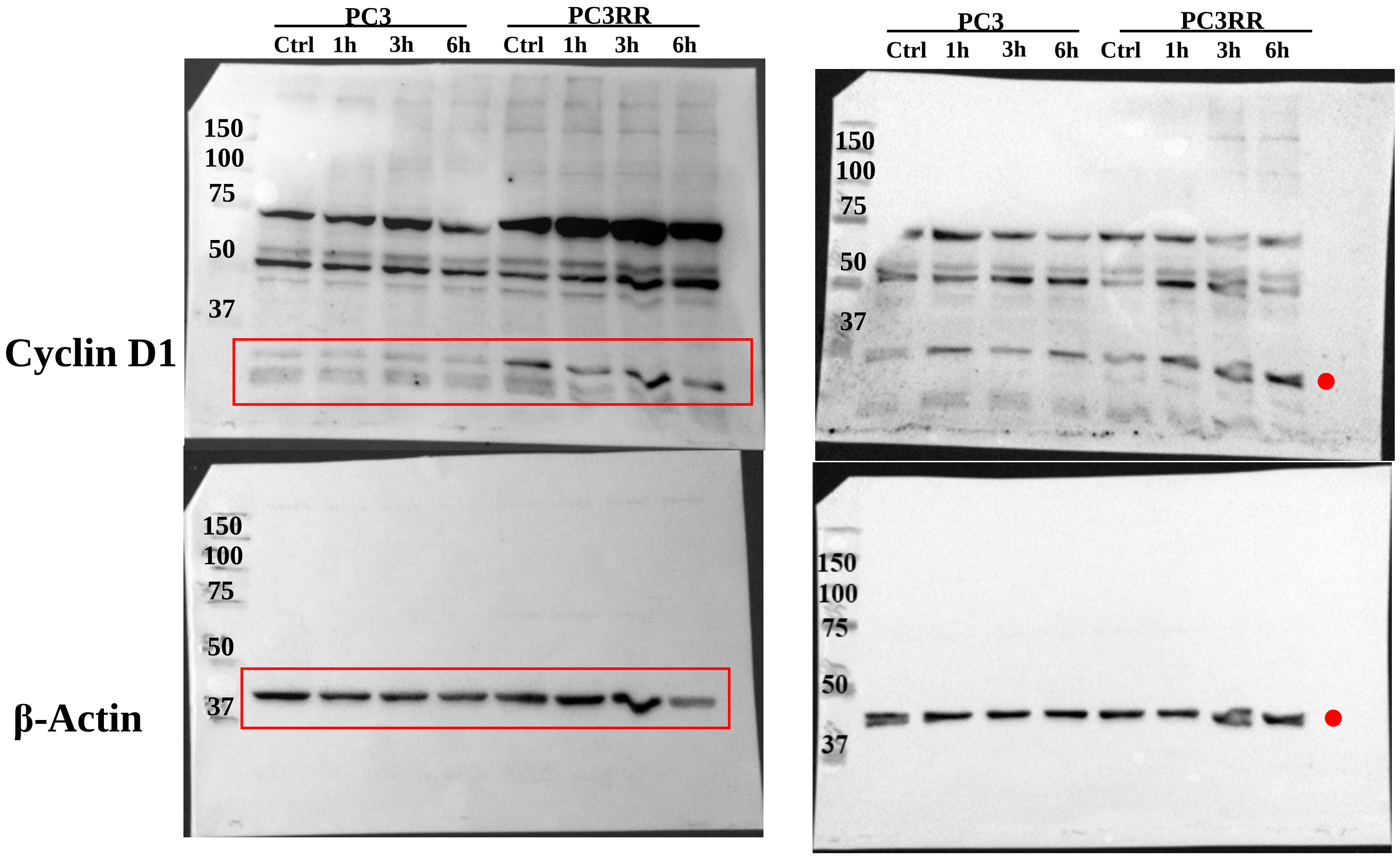
PC3
PC3RR
Ctrl 1h 3h 6h Ctrl 1h 3h 6h
150
100
75
50
37
Cyclin D1
β-Actin

Figure. S4A

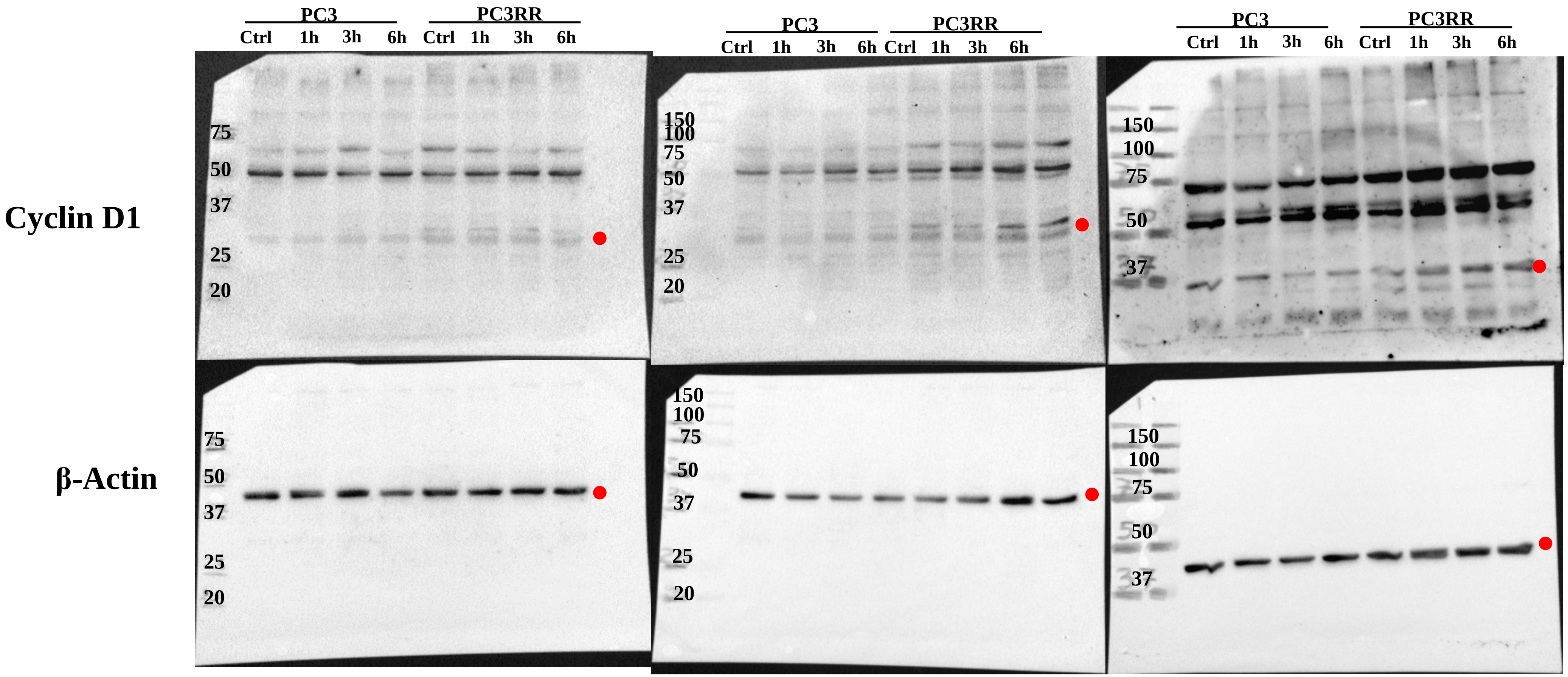

Figure. S4A bis

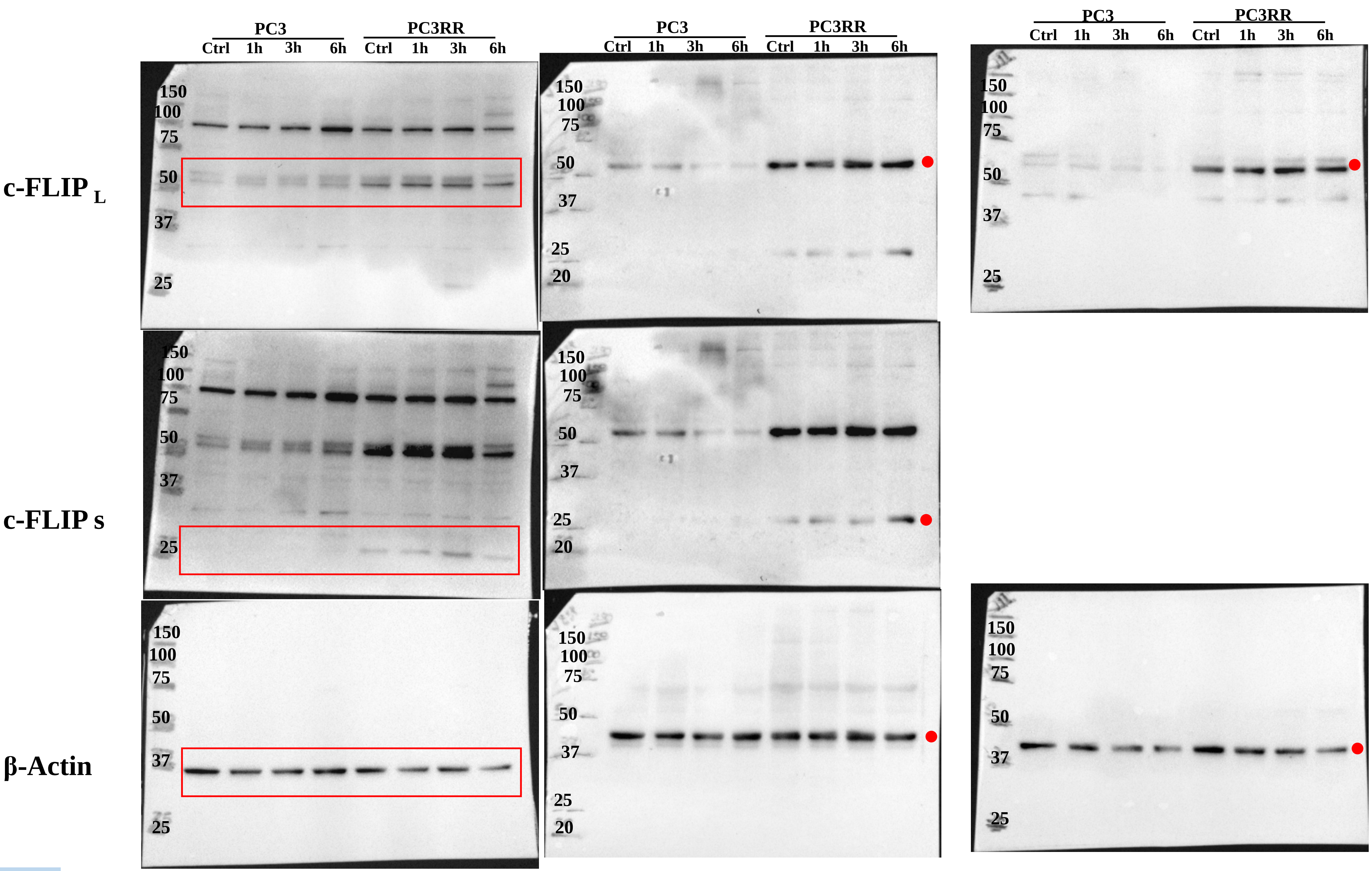

PC3
PC3RR
Ctrl
1h
3h
6h
150
100
75
50
37
25
20
c-FLIP L
c-FLIP s
β-Actin

Figure. S4B

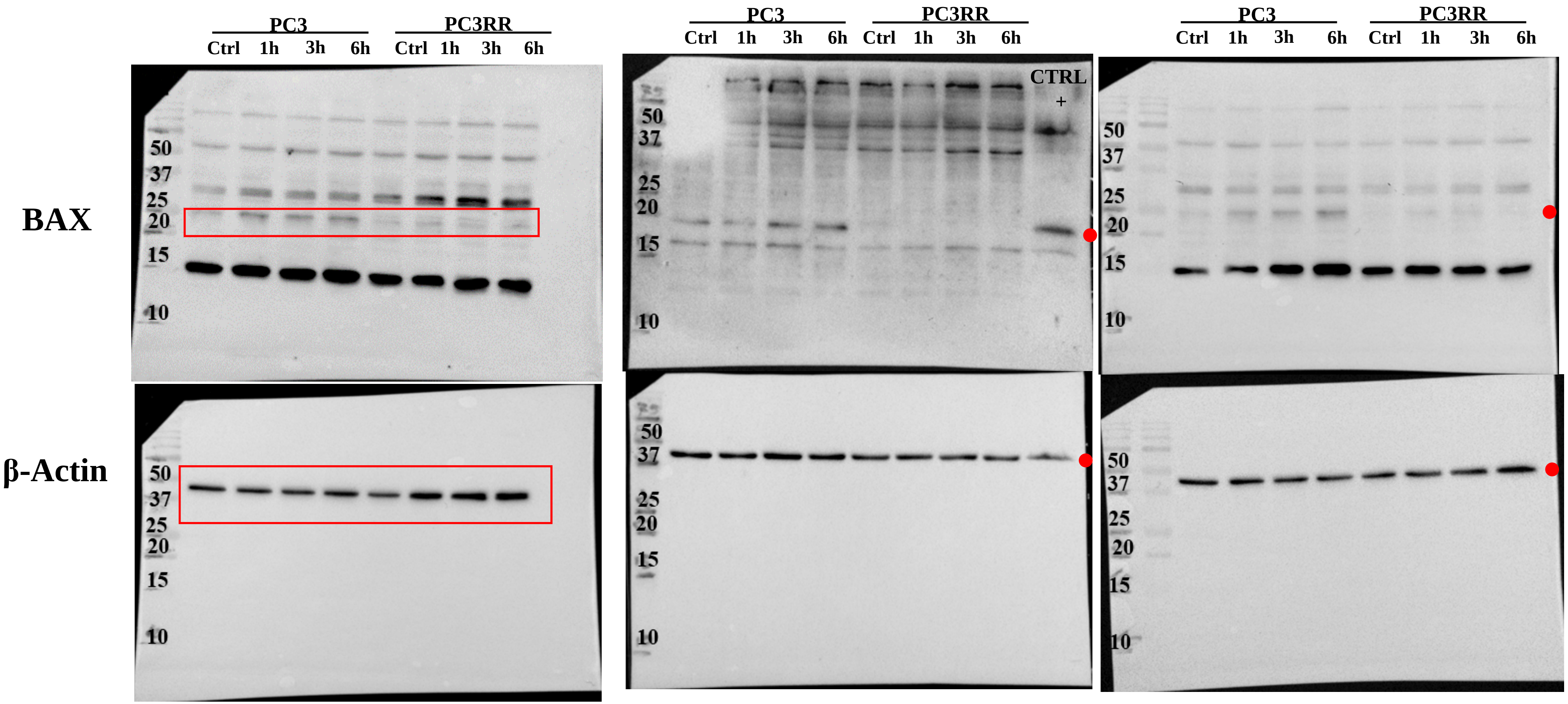

Figure. S4C

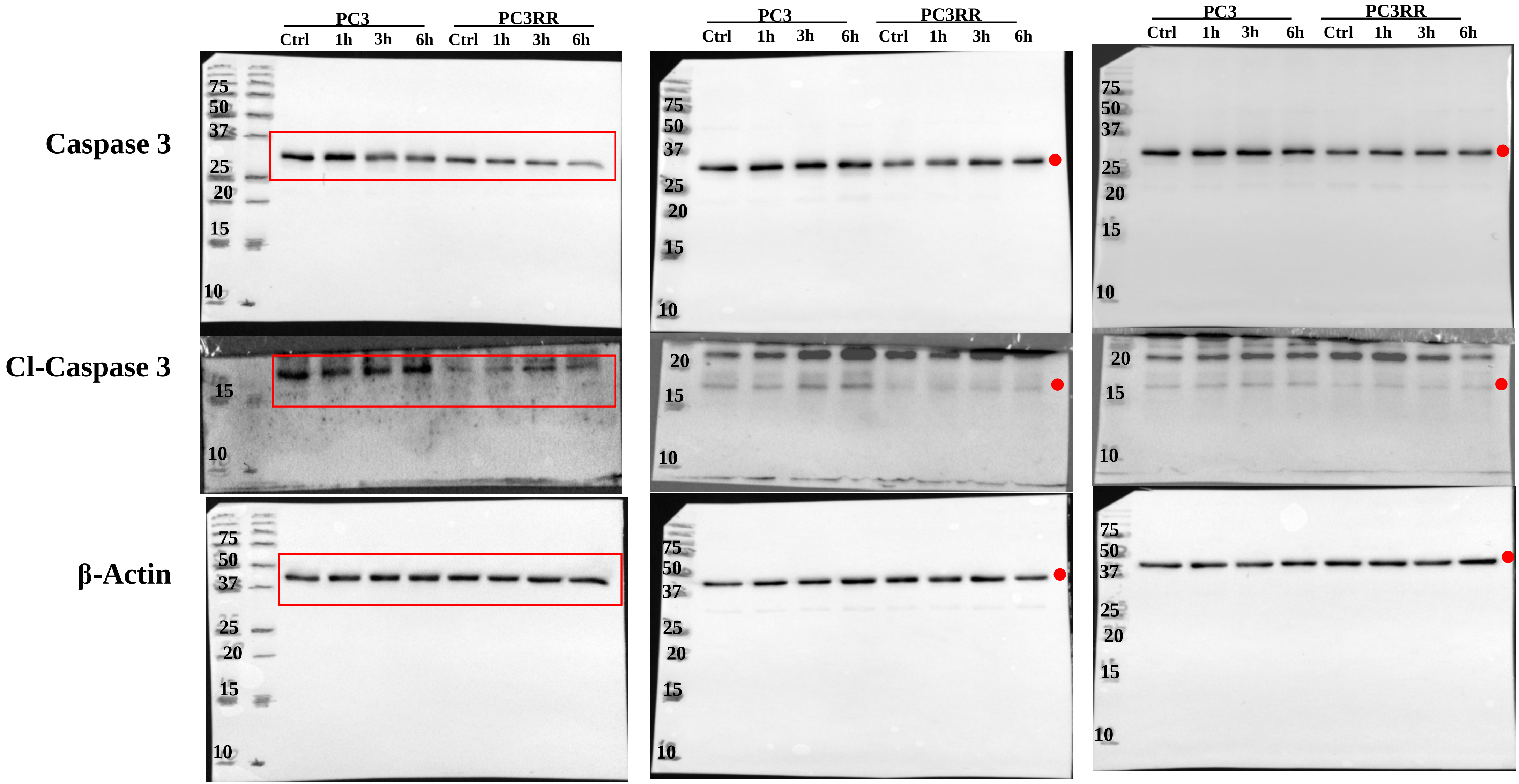
PC3
PC3RR
Ctrl
1h
3h
6h
Ctrl
1h
3h
6h
Caspase 3
Cl-Caspase 3
β-Actin
75
50
37
25
20
15
10

Figure. S4D

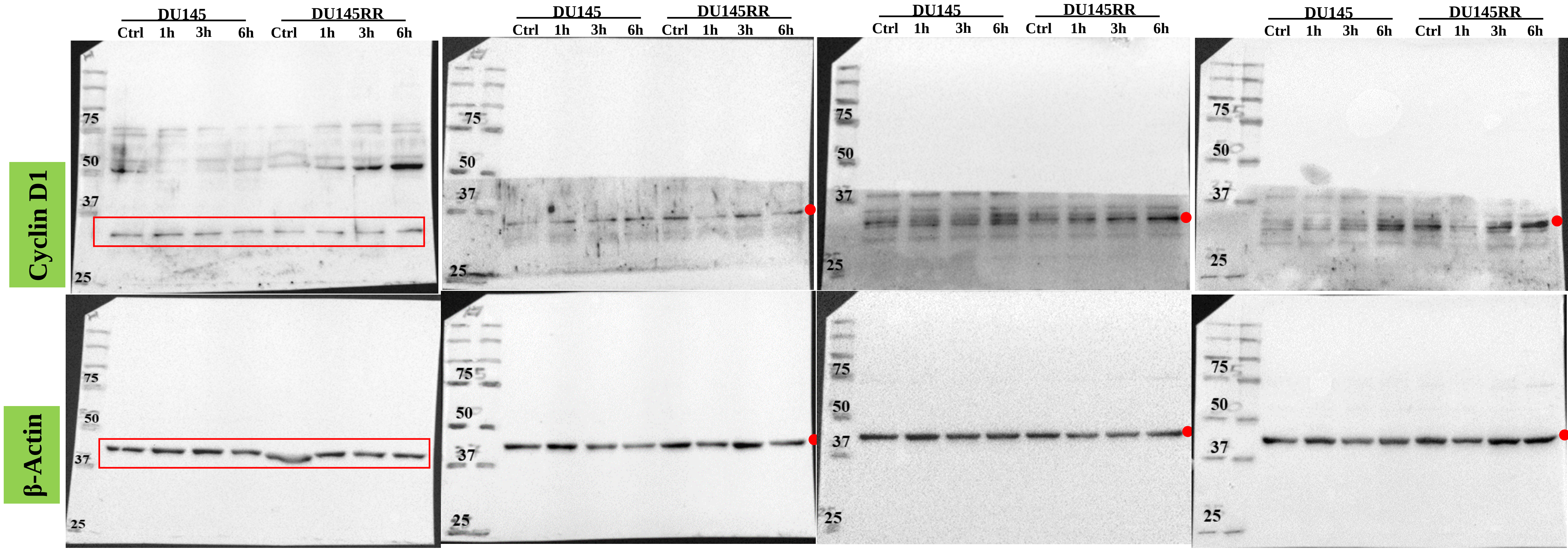
DU145
DU145RR
Ctrl
1h
3h
6h
Ctrl
1h
3h
6h
Cyclin D1
β-Actin
75
50
37
25

Figure. S4E

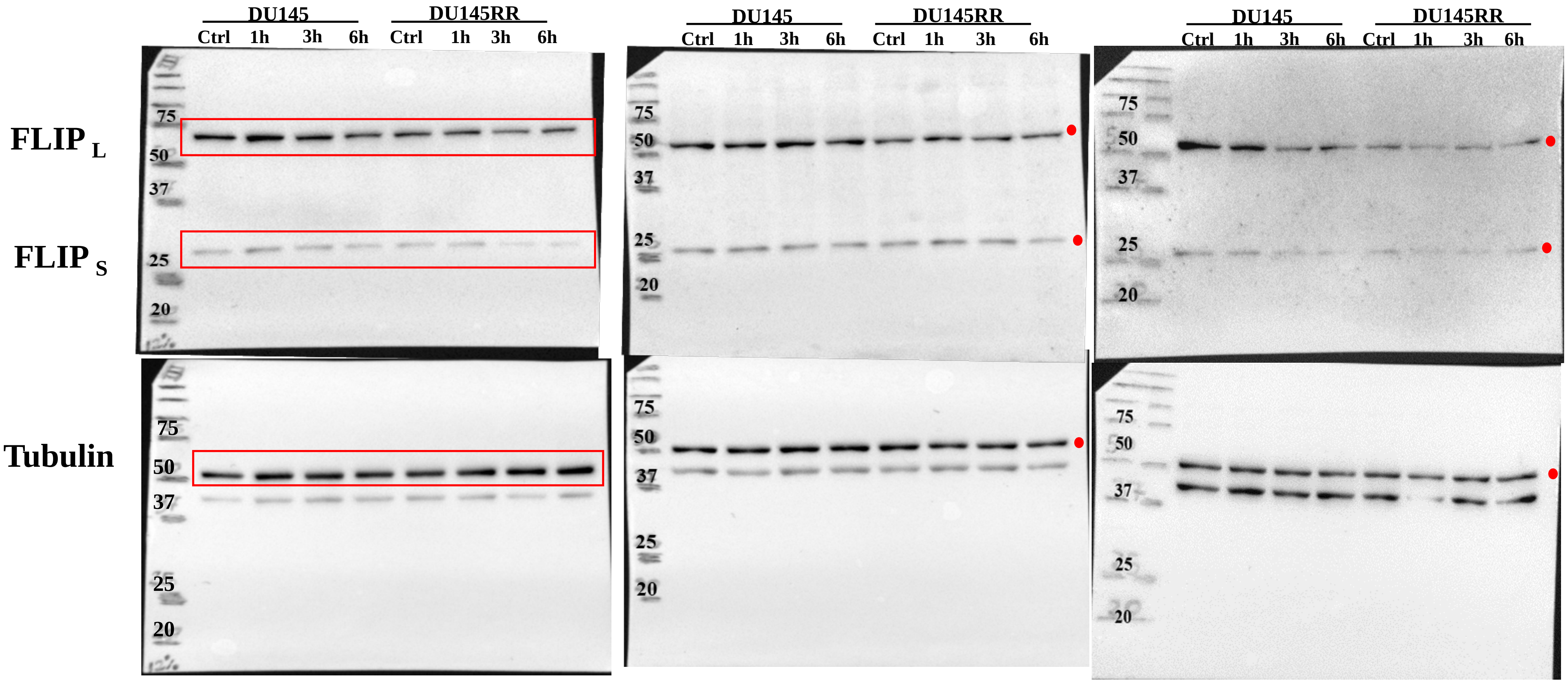
DU145
DU145RR
Ctrl 1h 3h 6h Ctrl 1h 3h 6h
FLIP L
FLIP S
Tubulin
75
50
37
25
20

Figure. S4F

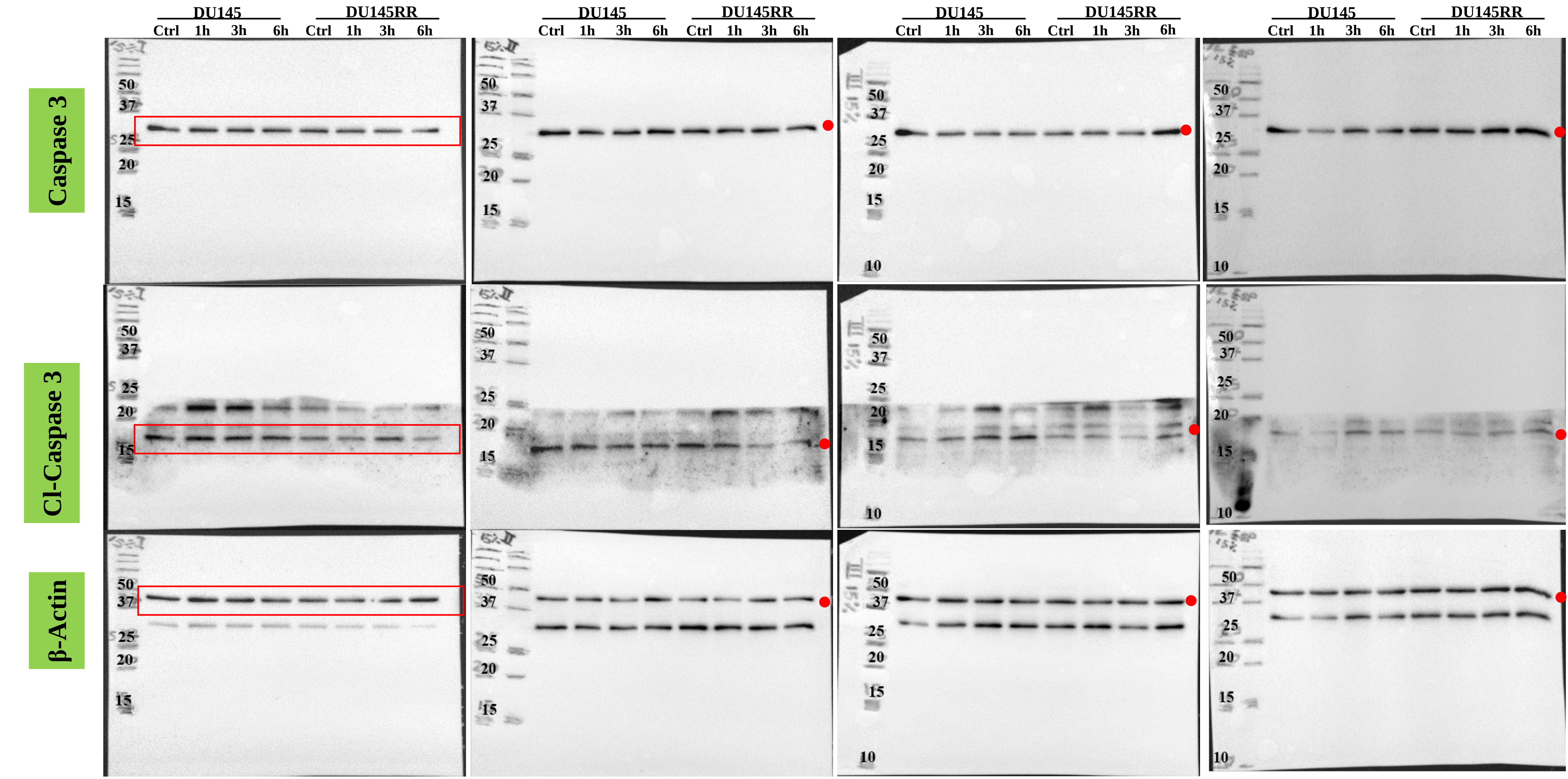
DU145
DU145RR
Ctrl 1h 3h 6h Ctrl 1h 3h 6h
Caspase 3
Cl-Caspase 3
β-Actin
50
37
25
20
15
10

Figure. S4G

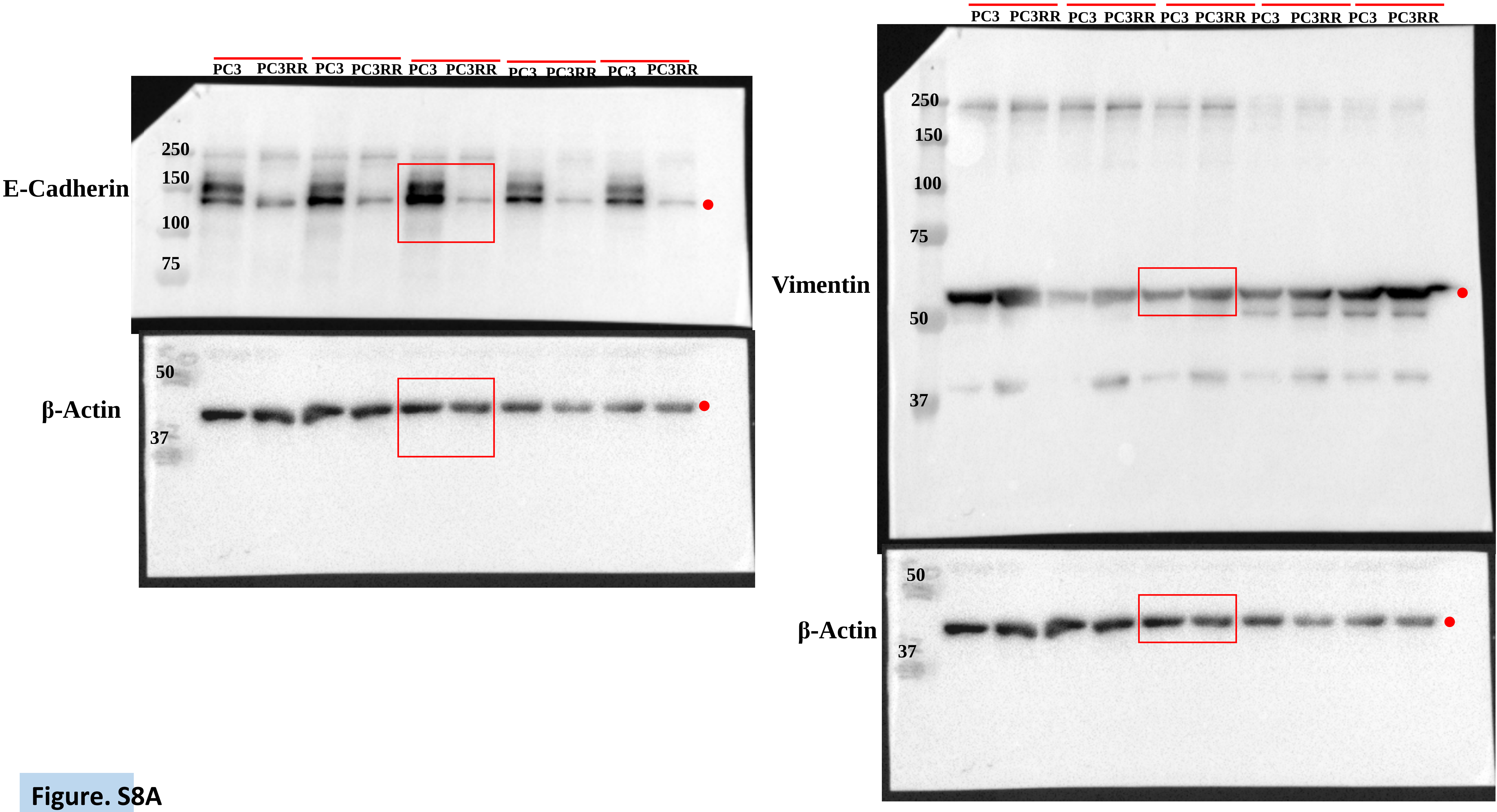
PC3 PC3RR PC3 PC3RR PC3 PC3RR PC3 PC3RR PC3 PC3RR
250
150
100
75
E-Cadherin
50
37
β-Actin
PC3 PC3RR PC3 PC3RR PC3 PC3RR PC3 PC3RR PC3 PC3RR
250
150
100
75
Vimentin
50
37
50
β-Actin
37

Figure. S8A

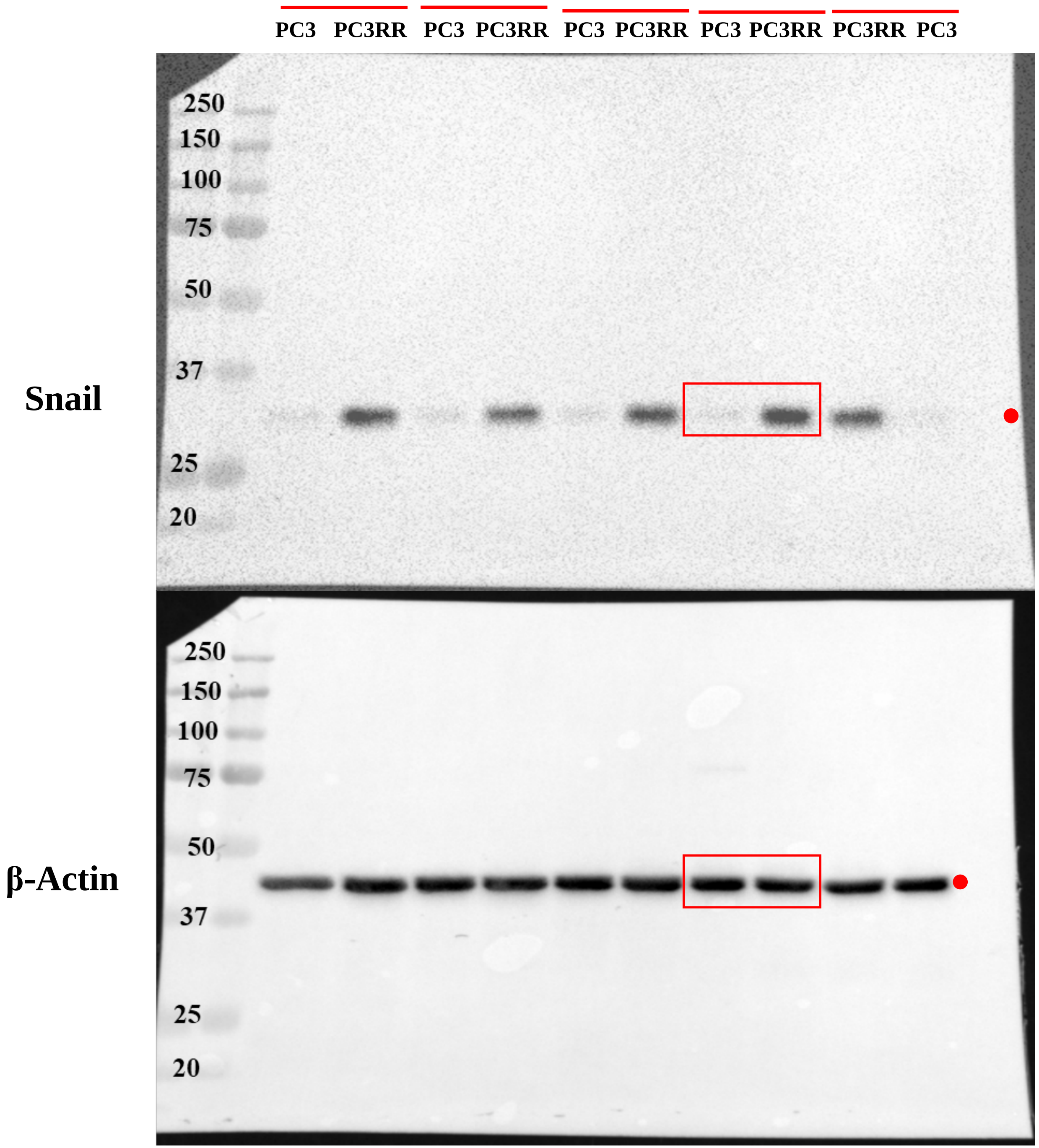
PC3 PC3RR PC3 PC3RR PC3 PC3RR PC3 PC3RR PC3RR PC3
Snail
250
150
100
75
50
37
25
20
β-Actin
250
150
100
75
50
37
25
20

Figure. S8A

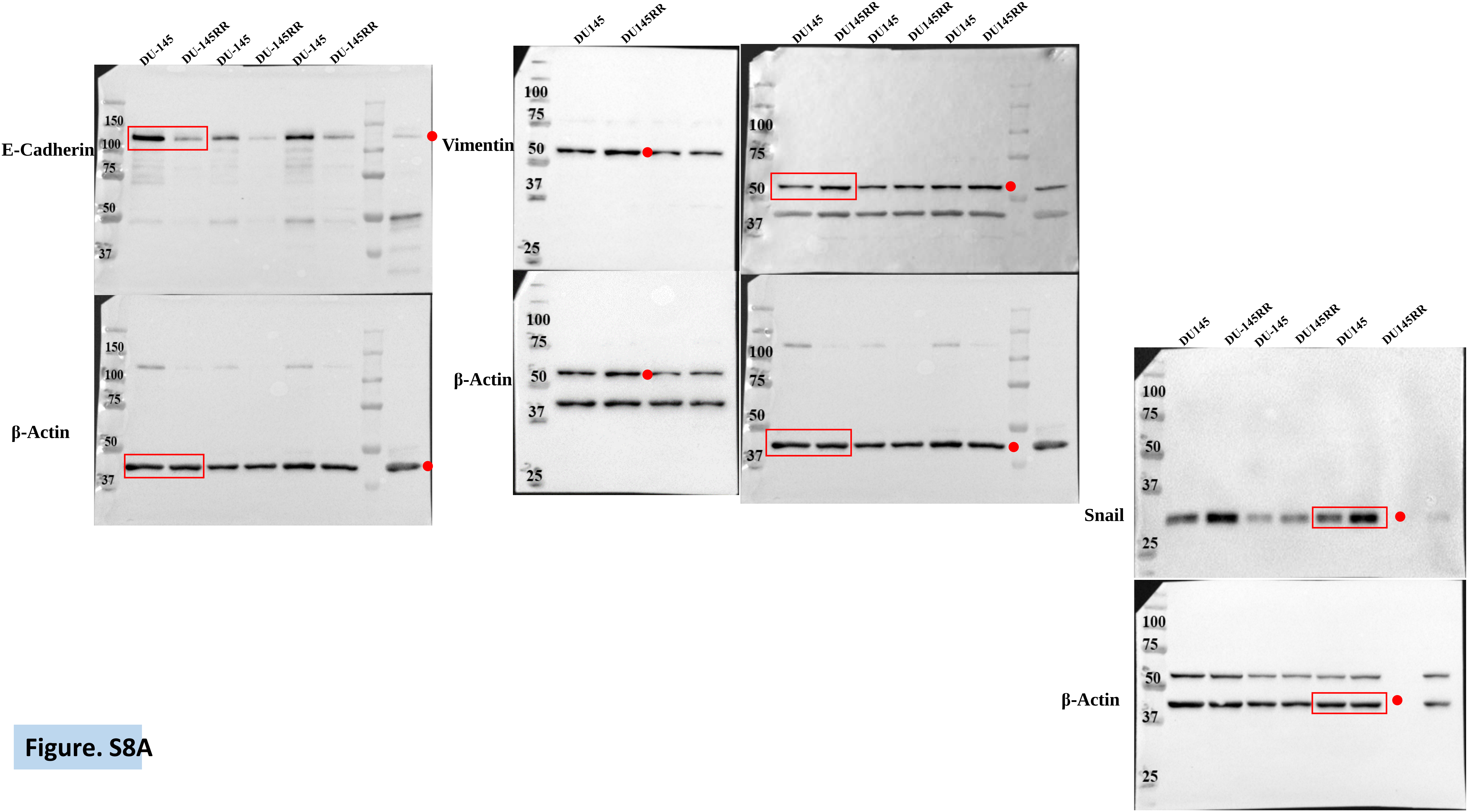
DU-145
DU-145RR
DU-145
DU-145RR
DU-145
DU-145RR
E-Cadherin
150
100
75
50
37
β-Actin
150
100
75
50
37
DU145
DU145RR
Vimentin
100
75
50
37
25
β-Actin
100
75
50
37
25
DU145
DU145RR
DU145
DU145RR
DU145
DU145RR
100
75
50
37
100
75
50
37
DU145
DU-145RR
DU-145
DU145RR
DU145
DU145RR
Snail
100
75
50
37
25
β-Actin
100
75
50
37
25

Figure. S8A

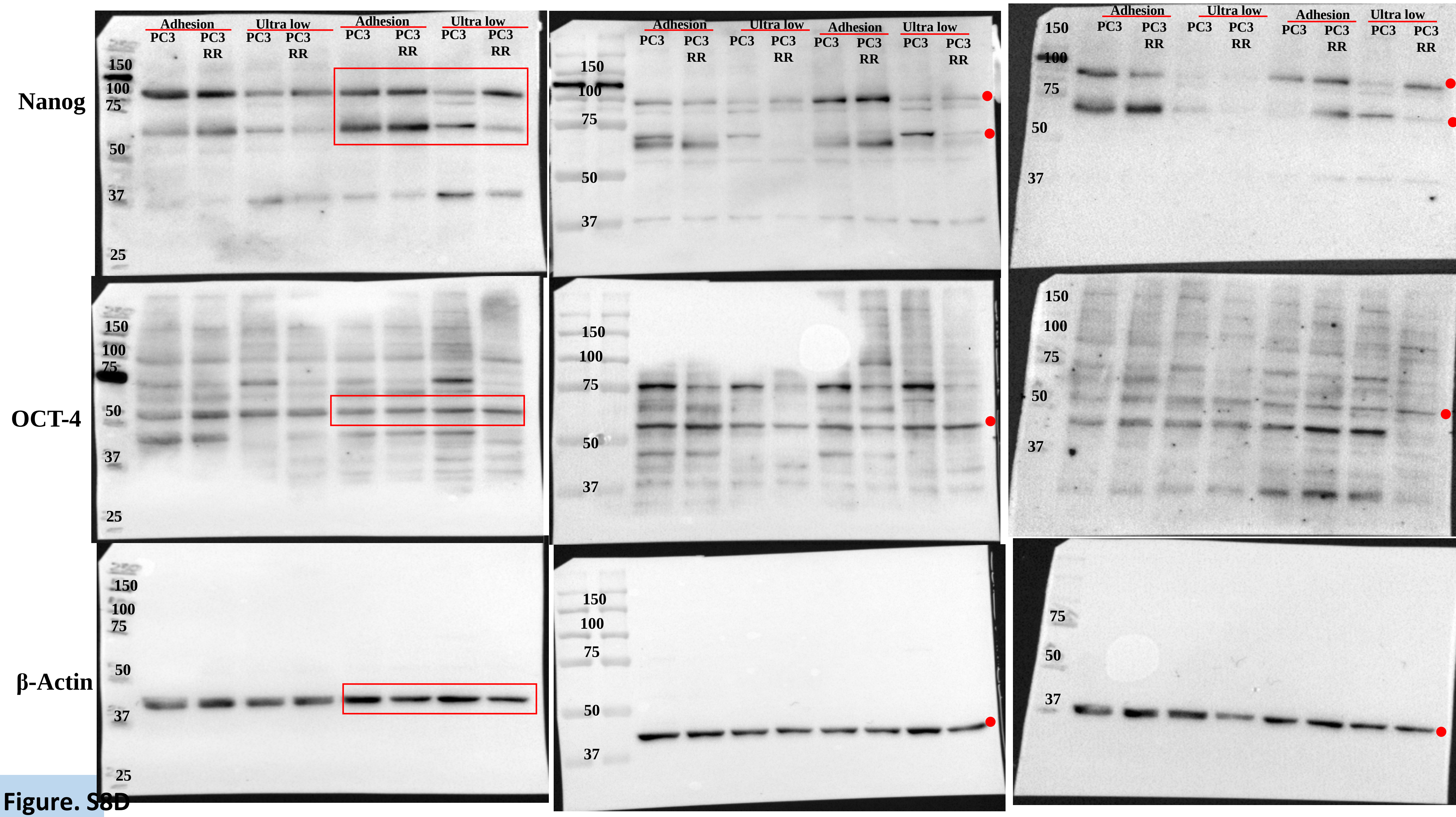

Nanog
OCT-4
β-Actin
Adhesion
Ultra low
PC3
PC3 RR
150
100
75
50
37
25

Figure. S8D

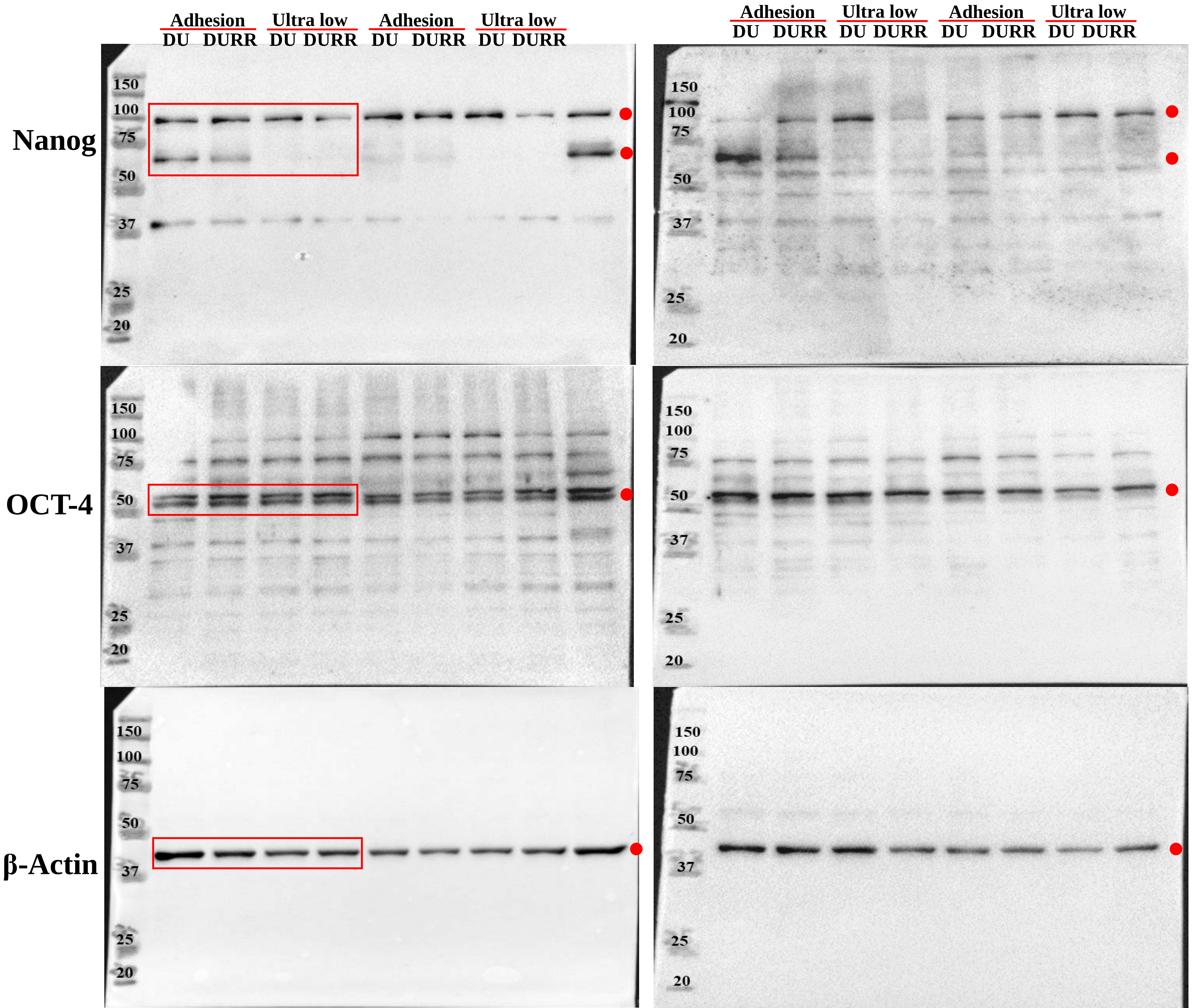

Adhesion
Ultra low
Adhesion
Ultra low
DU DURR DU DURR DU DURR DU DURR
Nanog
OCT-4
β-Actin
150
100
75
50
37
25
20

Figure. S8D

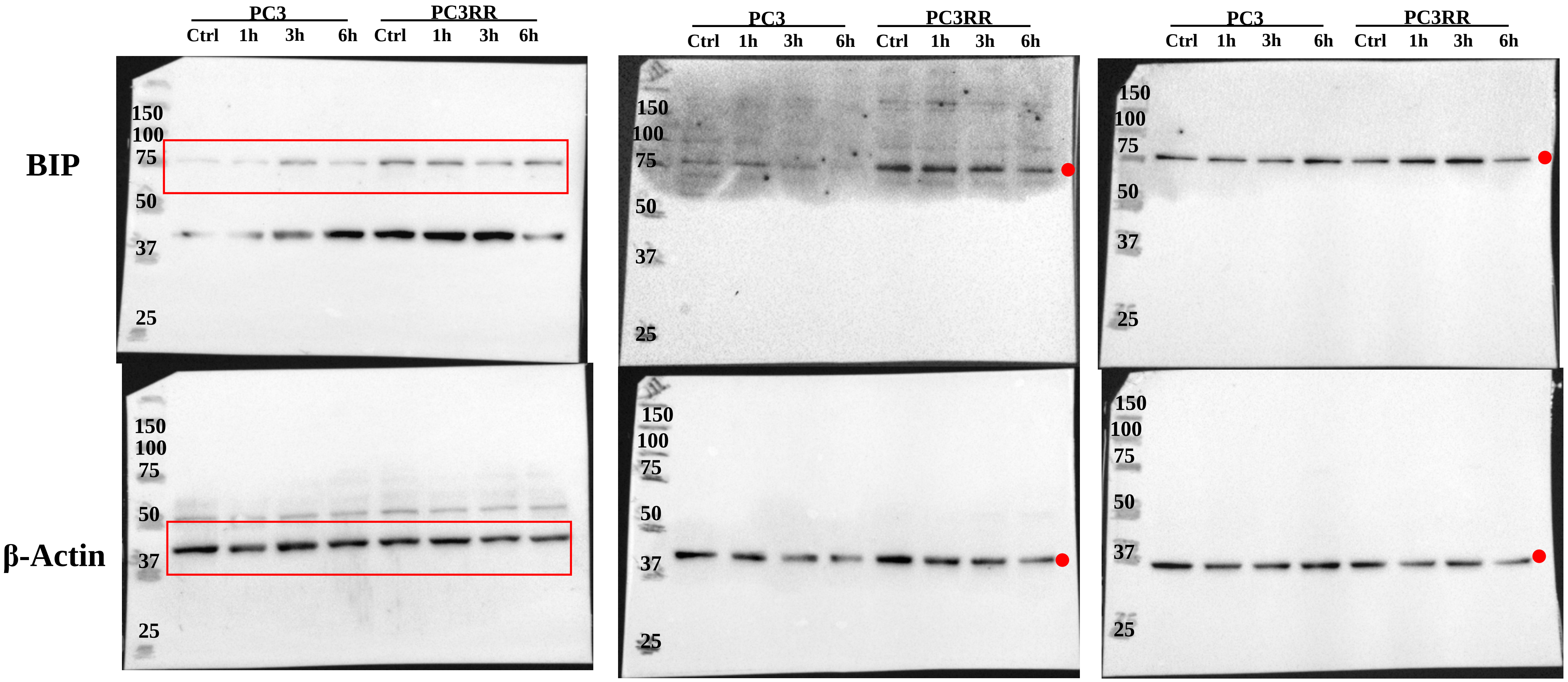

PC3
PC3RR
Ctrl
1h
3h
6h
Ctrl
1h
3h
6h
BIP
β-Actin
150
100
75
50
37
25

Figure. S9C

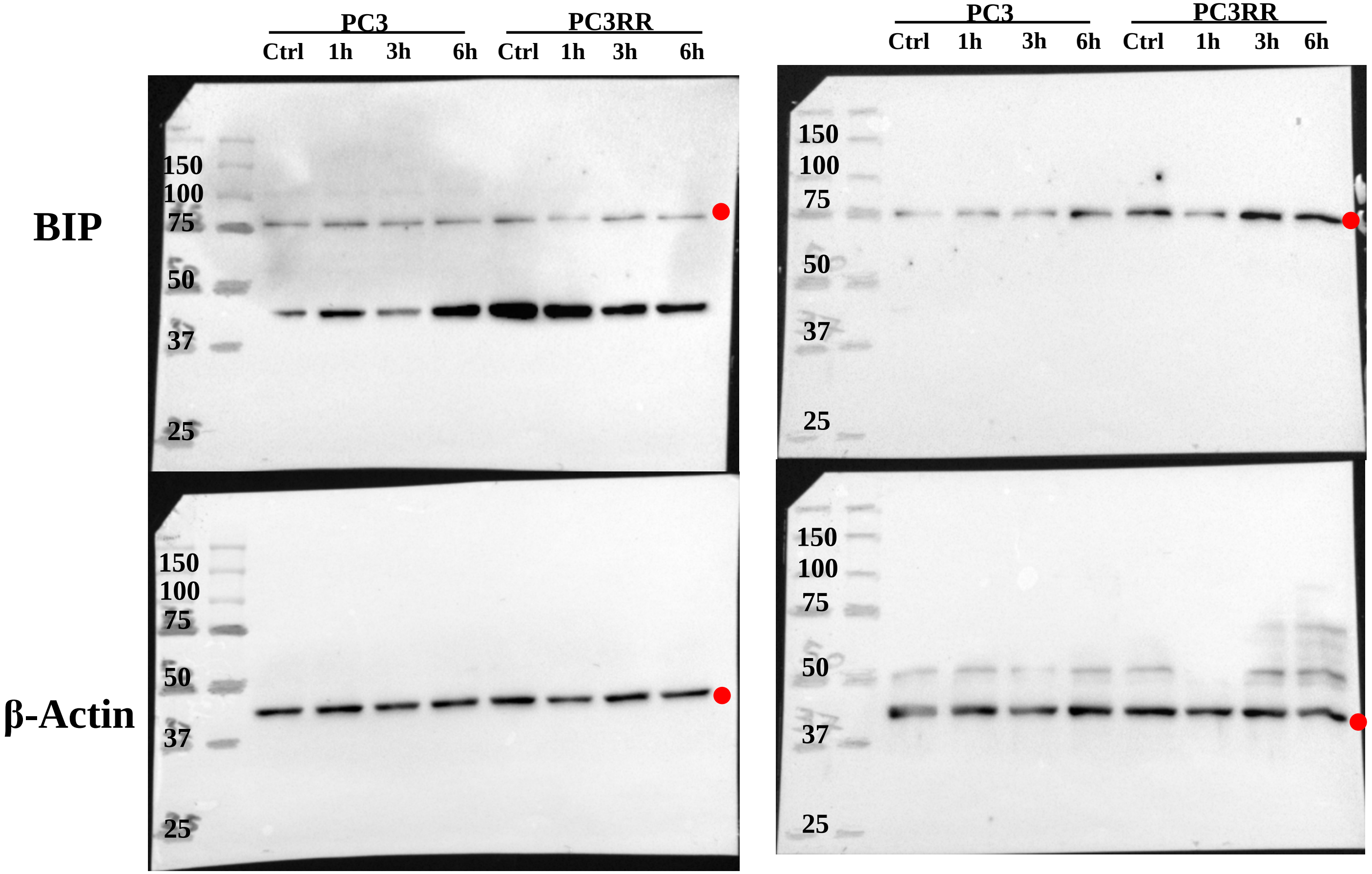

Figure. S9C bis

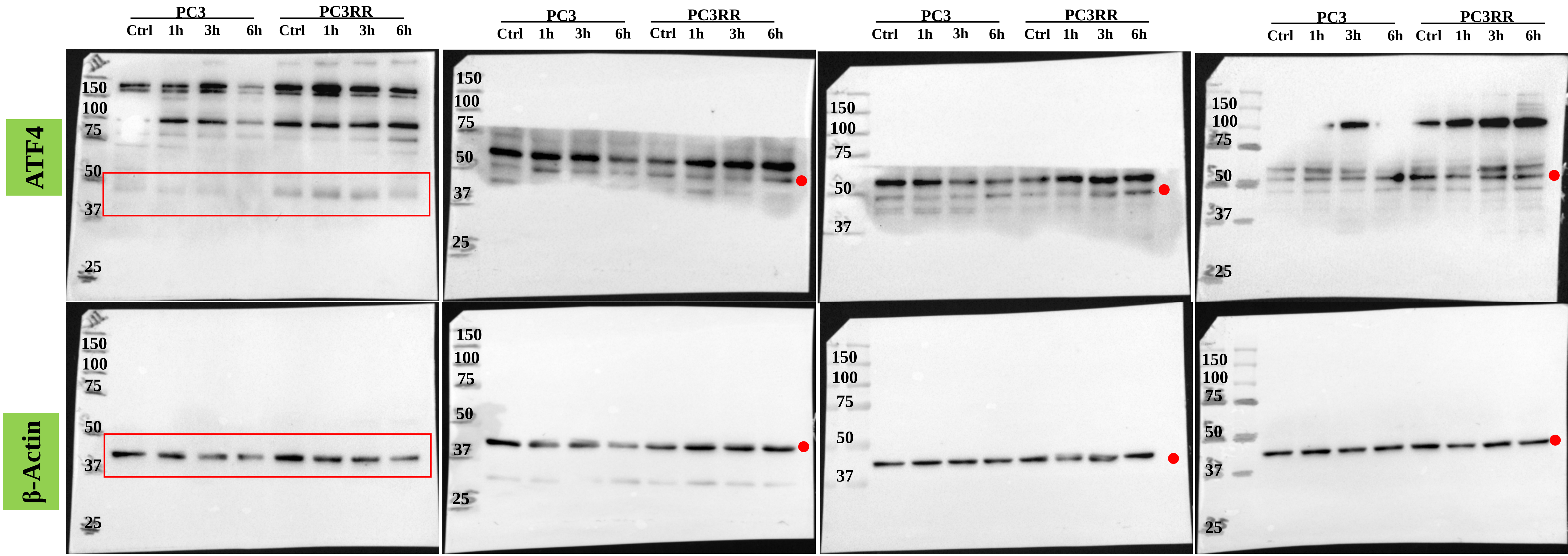

Figure. S9C

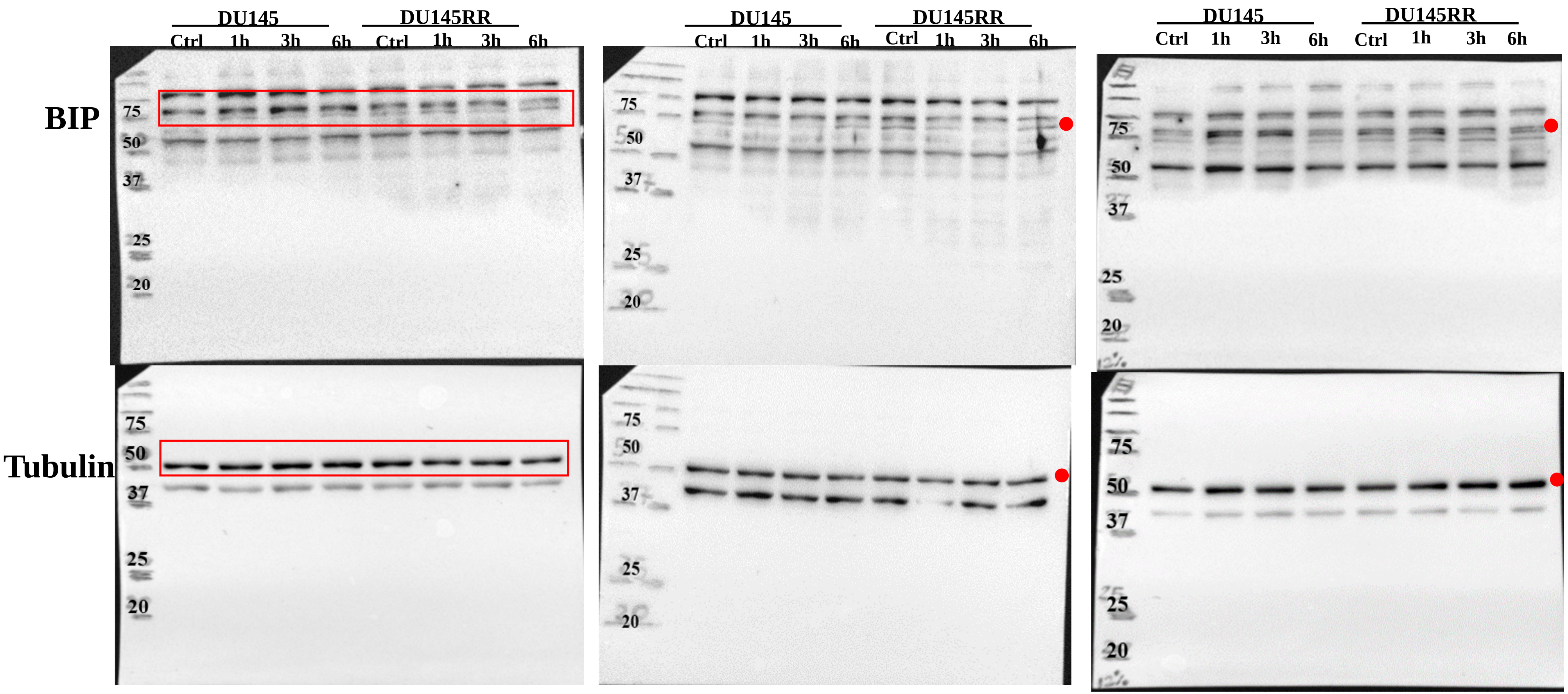

Figure. S9D

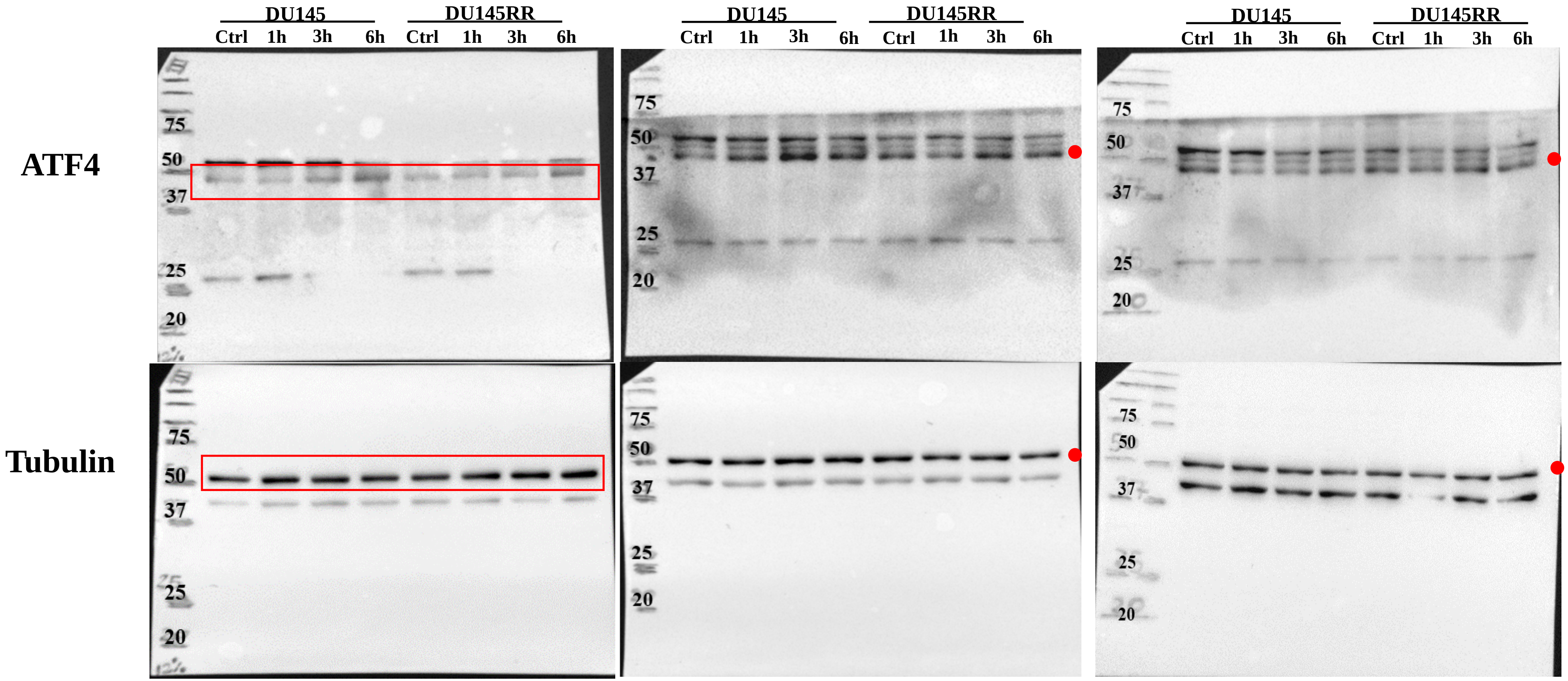

Figure. S9D

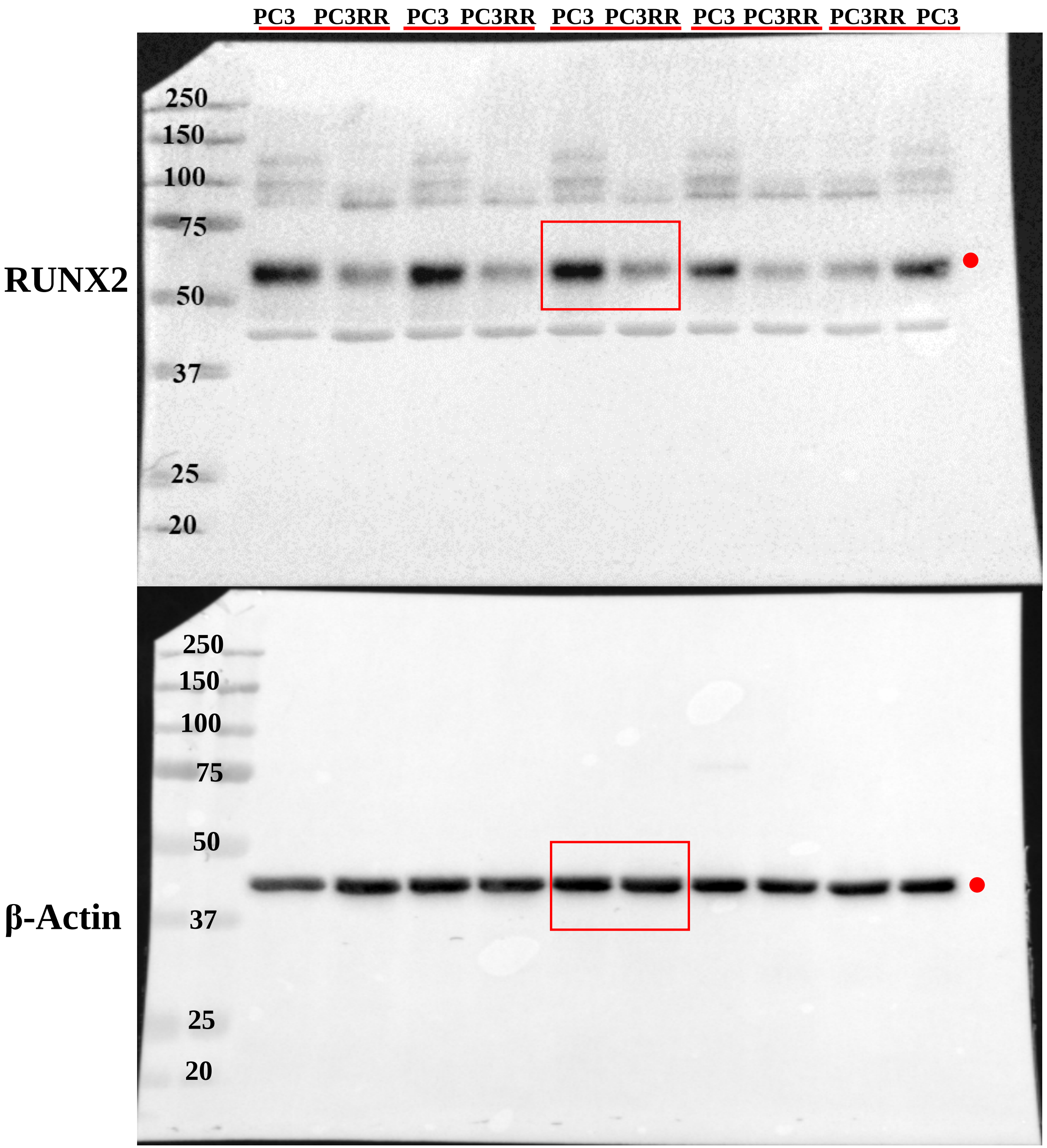
PC3 PC3RR PC3 PC3RR PC3 PC3RR PC3 PC3RR PC3RR PC3
RUNX2
250
150
100
75
50
37
25
20
β-Actin
250
150
100
75
50
37
25
20

Figure. S10
