# Supplementary material for: Radioresistance Mechanisms in Prostate Cancer Cell Lines Surviving Ultra-Hypo-Fractionated EBRT: Implications and Possible Clinical Applications
Source: Cancers (Basel). 2022 Nov 9;14(22):5504. doi: 10.3390/cancers14225504 (PMC9688510; doi:10.3390/cancers14225504)
Supplement: Supplementary file 1 [file cancers-14-05504-s001.zip › Supplementary Table S1 Sideri et al.pdf]

Table S1: List of primers used for gene expression analysis

Target gene	Forward primer	Reverse primer
Nanog	5'-TACCTCAGCCTCCAGCAGA -3'	5'-TTTTTGCGACACTCTTCTCTGC-3'
Sox2	5'-CACCCCTGGCATGGCTCTT-3'	5'-GAGCTGGCCTCGGACTTGA-3'
β-Actin	5'-TGCACCACACCTTCTACAATG-3'	5'-CAGCCTGGATAGCAACGTACAT-3'
IL-6	5'-ATGAACTCCTTCTCCACAAGC-3'	5'-GTTTTCTGCCAGTGCCTCTTTG-3'
CAT	5'-TCCTGAGAGAGTTGTGCATG-3'	5'-CTGCGATGGGAGTCTTCTTT-3'
CSTK	5'-CCCGCAGTAATGACACCCTT-3'	5'-TCGGGGATCTCTCTGTACCC-3'
GAPDH	5'-GGAATTTGGTCGTATTGGG-3'	5'-GGAAGATGGTGATGGGATT-3'
GPX4	5'-ATCGACGGGCACATGGTTA-3'	5'-ATCCGCAAACCACACTCAG-3'
NRF2	5'-GCAACAGGACATTGAGCAAGT-3'	5'-ACTTCTGTCAGTTTGGCTTCTG-3'
OPG	5'-TGGAATAGATGTTACCCTGTGTG-3'	5'-GCTGCTCGAAGGTGAGGTTA-3'
RUNX2	5'-TTACTTACACCCCGCCAGTC-3'	5'-TATGGAGTGCTGCTGGTCTG-3'
SOD2	5'-GTGAACAACCTGAACGTCAC-3'	5'-ATGACCACCACCATTGAACT-3'
EZH2	5'-TGTAAAATCAGAGTACATGCGA-3'	5'-TGACGATTGGAACTAAACAT-3'
