# Supplementary material for: Radioresistance Mechanisms in Prostate Cancer Cell Lines Surviving Ultra-Hypo-Fractionated EBRT: Implications and Possible Clinical Applications
Source: Cancers (Basel). 2022 Nov 9;14(22):5504. doi: 10.3390/cancers14225504 (PMC9688510; doi:10.3390/cancers14225504)
Supplement: Supplementary file 1 [file cancers-14-05504-s001.zip › Supplementary Table S2 Sideri et al.pdf]

Table S2

Percentages of necrotic and early/late apoptotic PC3 and PC3RR cells treated with Docetaxel for 24h and 48h.

24h	Docetaxel	Necrotic (%)	Early/late apoptotic (%)
PC3	Ctr	3.29 ± 0.63	1.91 ± 0.48
	10 nM	5.74 ± 2.38	2.37 ± 0.93
	20 nM	3.95 ± 1.2	2.31 ± 1.07
	50 nM	4.31 ± 1.09	2.75 ± 1.06
	100 nM	3.88± 0.96	2.54 ± 0.85
PC3RR	Ctr	5.79 ± 0.37	2.80 ± 1.35
	10 nM	5.82 ± 0.63	4.05 ± 0.70
	20 nM	7.03 ± 0.88	5.31 ± 0.94
	50 nM	10.1 ± 1.44	2.11± 0.23
	100 nM	11.1 ± 1.44	2.47 ±0.42

48h	Docetaxel	Necrotic (%)	Early/late apoptotic (%)
PC3	Ctr	4.06 ± 0.61	3.03 ± 0.7
	10 nM	5.11± 0.51	2.44 ± 0.12
	20 nM	4.52± 0.85	2.61 ±0.24
	50 nM	4.86±0.84	3.26 ±0.55
	100 nM	4.28±1.25	4.04 ±0.16
PC3RR	Ctr	5.66 ± 1.94	3.53 ± 1.34
	10 nM	7.85 ± 1.42	3.69 ± 0.44
	20 nM	9.36 ±1.91	5.5 ± 0.74
	50 nM	13.89 ± 2.02	12.97 ± 2.74
	100 nM	19.78 ± 4.78	14.42 ± 1.30
